# Tumor-derived EV miRNA signatures surpass total EV miRNA in supplementing mammography for precision breast cancer diagnosis

**DOI:** 10.7150/thno.99245

**Published:** 2024-10-07

**Authors:** Young Kim, Jee Ye Kim, Sol Moon, Hyojung Lee, Suji Lee, Joon Ye Kim, Min Woo Kim, Seung Il Kim

**Affiliations:** Department of Surgery, Yonsei University College of Medicine, Seoul 03722, Republic of Korea

**Keywords:** breast cancer, early diagnosis, mammography, circulating biomarkers, extracellular vesicles (EVs), microRNA (miRNA), immunoaffinity capture, liquid biopsy, tumor progression, diagnostic accuracy

## Abstract

**Background:** With the rising global incidence and mortality rates of breast cancer, early diagnosis is becoming increasingly crucial. The World Health Organization (WHO) recommends mammography as a primary screening tool. However, despite its clinical benefits, mammography has potential risks including radiation exposure, unnecessary follow-up, and overdiagnosis due to false positives, particularly in cases of early cancer or dense breast tissue. In this study, we aimed to address these concerns by introducing an innovative diagnostic method that employs circulating biomarkers to enhance the existing screening techniques

**Methods:** Breast cancer-derived extracellular vesicles (BEVs) were isolated from the bloodstream using advanced immunoaffinity capture techniques. Subsequently, we analyzed the microRNA (miRNA) profiles of BEVs in plasma samples from 120 patients with breast cancer, 46 with benign tumors, and 45 healthy controls.

**Results:** This retrospective study identified a distinct signature of five EV miRNAs (miR-21, miR-106b, miR-181a, miR-484, and miR-1260b) that effectively differentiated patients with breast cancer from healthy controls. This signature provides essential insights into tumor progression, metastasis, and the risk of recurrence. Notably, overexpression of this signature correlated with poorer survival outcomes.

**Conclusions:** Our novel gene signature-based approach not only complements existing diagnostic methods with high accuracy but also provides a deeper understanding of the molecular aspects of breast cancer, heralding a significant advancement in precision medicine and personalized cancer care.

## Background

Breast cancer is the most common cancer among women worldwide, with increasing incidence and mortality rates each year 1. The World Health Organization recommends mammography as the primary screening tool for early stage breast cancer aimed at prompt treatment and reduced morbidity and mortality 2. However, current mammography techniques have limitations 3; apart from providing essential clinical benefits, it also poses risks such as radiation exposure, unnecessary follow-up, and overdiagnosis due to false-positive results 4. Particularly, dense breast tissue can hinder early diagnosis by reducing the sensitivity of mammograms and obscuring the visualization of early cancers 5, 6. Despite advancements in digital mammography systems, clinical sensitivity remains relatively low, particularly for dense breast tissue, which hovers at approximately 61.5%. Combining breast ultrasound with mammography increases this rate to approximately 81.5% 7-10.

Radiological diagnoses can identify tumors but often miss their detailed characteristics. Although incorporating artificial intelligence boosts diagnostic accuracy, it fails to capture the molecular and genetic complexities of tumors 11. In traditional biopsies, samples obtained from solid biopsies or surgical excisions represent only a portion of the tumor and fail to account for spatial and temporal heterogeneity 12. Moreover, they pose challenges such as potential complications, contamination risks, and human error 13, 14. In comparison, circulating biomarkers in the bloodstream provide a broader view of a tumor's molecular characteristics 15. Notably, exosomes that are naturally released into the blood are emerging as the prime focus for liquid biopsy methods 16. However, these methods require effective isolation of tumor-related exosomes from the blood.

As primary tumors evolve, their microRNA (miRNA) expression undergoes dynamic changes, particularly within exosomes. These miRNA alterations have implications both within the tumor microenvironment and at distant locations, playing pivotal roles in tumor progression, tissue invasion, angiogenesis, metastatic niche formation, and evasion of immune surveillance 17. Importantly, miRNA profiles often show high similarity in relevant tumor tissues and the corresponding exosome samples, highlighting the diagnostic potential of exosome analysis 18. However, current exosome isolation methods, such as ultracentrifugation and ultrafiltration, are labor-intensive, inefficient, and lack the specificity needed to isolate tumor-derived exosomes 19. To overcome these drawbacks, several recent studies have focused on immunoaffinity capture techniques that exploit the unique surface epitopes of cancer cells for the selective purification of tumor-derived exosomes 20-23. This method has the following merits: (1) Immunocapture employs antibodies that specifically bind to tumor-specific antigens present on the surface of EVs. This allows for the selective enrichment of EVs; (2) immunocapture can detect low-abundance EVs in biological fluids and boost the sensitivity of downstream analyses, providing critical insights into disease states such as early-stage cancers; and (3) Compared to other EV isolation methods, such as ultracentrifugation or size-exclusion chromatography, immunocapture typically results in higher purity of the isolated EVs in a shorter time.

In recent years, the emergence of liquid biopsies for precision medicine has led to remarkable advancements in cancer diagnostics, gaining substantial interest in the medical field. By leveraging this evolution, in this study, we developed an innovative diagnostic technique to improve the efficiency of the existing liquid biopsy technologies while addressing the limitations of conventional mammography. Our strategy focused on identifying unique markers on vesicle surfaces, enabling the isolation of tumor-derived exosomes. This approach offers vital clues regarding the underlying characteristics of cancer. By targeting these specific markers, we can accurately identify vesicles associated with breast cancer and delve deeply into the complex miRNA patterns. In conclusion, this study effectively enhanced the diagnostic accuracy and depth, providing clinicians with great insights for breast cancer diagnosis.

## Methods

### Patient selection and plasma sampling

Informed consent for the use of plasma samples for research purposes was obtained from all participants. Clinical samples were obtained from patients who visited Severance Hospital (Seoul, South Korea) according to the guidelines of the Independent Ethics Committee of Yonsei University College of Medicine (IRB approval no. 4-2020-1292; approval date January 4, 2021; Seoul, South Korea). Preoperative plasma samples were collected from 120 patients with BC, 45 healthy women, and 46 patients with benign tumors who visited Severance Hospital between May 2010 and August 2021 and were retrospectively registered in the present study. Clinical information was retrospectively gathered from the Electronic Medical Records (EMR) system at Severance Hospital.

To qualify for providing the clinical sample, subjects were required to meet the following criteria: (1) confirmed pathological diagnosis of BC, (2) no chemotherapy or radiotherapy prior to blood collection, (3) an assessment for hemolysis before the extraction of EVs to ensure the quality of the plasma samples, and (4) the exclusion of patients with a history of other malignancies or existing medical conditions. Blood was collected into EDTA tubes (0.02%) and centrifuged at 1500 ×*g* for 15 min. Then, the supernatant plasma was stored at -80 °C. For EV isolation, plasma samples were thawed and pre-clarified by centrifugation at 2000 ×*g* for 10 min at 4º C and then at 10,000 ×*g* for 30 min. Filtration through a 0.22 µm Millipore filter (cat. SLGPR33RB; Merck Millipore Ltd., Billerica, MA, USA) was then performed, and a 200 µL aliquot of purified plasma was used for further EV isolation step.

### Cell culture and EV harvest

Breast cell lines used in this study reflected representative breast tumor subtypes: MCF-10a for benign breast tissue (human mammary epithelial cells); MCF7 for luminal A subtype; BT-474 for luminal B subtype; SK-BR-3 for HER2 subtype; and MDA-MB-453, HCC1187, MDA-MB-468, HCC70, HCC1937, MDA-MB-231, and Hs578T for triple-negative breast cancer (TNBC) subtype. All cell lines were procured from American Type Culture Collection; Manassas, VA, USA. All cell lines were grown in Roswell Park Memorial Institute-1640 medium (RPMI-1640; cat. 22400-089) supplemented with 10% fetal bovine serum (FBS; cat. 12483-020) and 1% penicillin-streptomycin (cat. 15140-122) (all from Gibco; Thermo Fisher Scientific Inc., Waltham, MA, USA). All cells were grown as monolayer cultures and maintained in a humidified atmosphere of 5% CO_2_ at 37 °C.

For cell line-derived EV harvesting, breast cell lines were grown to 70-80% confluency in RPMI media with 10% FBS. The Media were removed, and the cells were rinsed three times with phosphate-buffered saline (PBS) and grown in serum-depleted media. After 72 h incubation at 37 °C with 5% CO_2_, the EV-enriched media were harvested and centrifuged once at 600 ×*g* for 30 min to eliminate the cells. The EVs were further concentrated from the cell-free supernatants using a Macrosep Advance Centrifugal Device (cat. MAP100C37, 100 K, Pall Life Science, Port Washington, NY, USA).

### Optimization of BEV isolation process

Before profiling and isolating BEVs, we established an ideal antibody-to-magnetic bead (Ab-to-Mg bead) ratio and determined the necessary conditions with MDA-MB-231 EV, a representative breast cancer cell line EV, samples for effective EV capture. First, the DiD staining solution from the Vybrant™ Multicolor Cell Labeling Kit (cat. V22889; Thermo Fisher Scientific Inc.) was mixed with MDA-MB-231-derived EV samples. After staining according to the manufacturer's protocol, the samples underwent magnetic separation to isolate the BEVs under various conditions, including Ab-to-Mg bead ratios, incubation times, and temperatures. The absorbance spectra of the DiD-stained EVs were then measured using a spectrophotometer microplate reader (Infinite 200 PRO; Tecan Group Ltd, Männedorf, Switzerland) across a wavelength range of 400 nm to 800 nm. The resulting absorbance spectra were analyzed to identify the characteristic peaks of the DiD dye, which provided insights into the effectiveness of BEV isolation under various tested conditions. The optimal BEV isolation condition was determined based on the highest absorbance observed at the wavelength corresponding to the maximum absorption of DiD dye, typically around 650 nm. The optimal antibody-to-Mg bead ratio was determined to be 1:80 (weight/weight), with the most effective incubation time of 2 h at 25 °C, as depicted in **Figure S1**.

### Isolation of BEVs

EV isolation in this study was categorized into BEV isolation and total EV (TEV) isolation, with TEVs serving as comparable controls for BEVs. TEVs were isolated from 200 μL of plasma using a commercial precipitation-based Total Exosomes Isolation Kit (cat. 4484450; Thermo Fisher Scientific Inc.), according to the manufacturer's protocol. This polyethylene glycol (PEG) precipitation method can co-isolate other components, such as other types of EVs, protein aggregates, lipoproteins, and free nucleic acids, making it less specific. Therefore, we focused on BEVs as a more tumor-specific EV isolation method.

For BEV isolation, we utilized 200 µg of 3-μm streptavidin-labeled magnetic beads conjugated with 2.5 µg of biotinylated antibodies targeting the following breast cancer surface markers: epithelial cell adhesion molecule (EpCAM), glypican-1 (GPC-1), CD49b (integrin α2), CD51 (integrin αv), and CD49f (integrin α6). Using this optimized method, we evaluated EV surface markers for effective BEV isolation through flow cytometry (FACS LSR Fortessa System, Becton Dickinson, Franklin Lakes, NJ, USA). Immunobeads labeled with each surface marker were prepared and incubated with EVs derived from various breast cell lines, including MCF-10a, SK-BR-3, MCF7, BT-474, MDA-MB-453, HCC1187, MDA-MB-468, HCC70, HCC1937, MDA-MB-231, and Hs578T, for 2 h at 25 °C. The samples were then rinsed twice with PBS to prevent nonspecific binding. Subsequently, they were incubated with 5 µL of anti-CD63-PE-Cy7 antibodies (cat. 561982; Becton Dickinson), targeting the EV-specific marker, in the dark for 30 min at 4 °C for the relative quantification of BEVs.

Comprehensive flow cytometry analysis identified EpCAM, CD51, and CD49b as the most effective surface markers for isolating BEVs that accurately reflect the characteristics of the originating breast tumors. These markers were subsequently used in the analysis of clinical samples in this study.

### Visualization of BEVs

For scanning electron microscopy (SEM), immunobeads bound to BEVs were fixed for 24 h in Karnovsky's fixative consisting of 2% glutaraldehyde (cat. 354400; Merck KGaA, Darmstadt, Germany) and 2% paraformaldehyde (cat. 818715; Merck KGaA) dissolved in 0.1 M phosphate buffer (pH 7.4). Following fixation, the samples were washed twice for 30 min with 0.1 M phosphate buffer to remove any residual fixative. The beads were then post-fixed in 1% osmium tetroxide (OsO_4_) for 2 h, which helped preserve the samples by stabilizing the lipid membranes and imparting contrast for electron microscopy. The samples were then dehydrated using a gradually ascending ethanol series (50-100%) with a Critical Point Dryer (cat. CPD300; Leica Microsystems, Wetzlar, Germany) and coated with platinum using ion sputtering (cat. ACE600; Leica Microsystems). The samples were then examined under a scanning electron microscope (Carl Zeiss AG, Oberkochen, Germany, model MERLIN) at a magnification of ×10,000 to allow a detailed visualization of the BEV morphology.

For confocal microscopy, immunobeads bound to BEVs were incubated with 5 µL of anti-CD63-PE-Cy7 antibodies (cat. 561982; Becton Dickinson) for 1 h at room temperature in the dark to facilitate detection. Fluorescence images were captured using a confocal microscope equipped with a 562 nm laser (LSM 780; Carl Zeiss AG). Z-stack images were collected with a step size of 0.2 µm, covering a total depth of 5 µm. The resulting z-stack images were processed and reconstructed using ZEN blue software to visualize the 3D structure of the labeled EVs on the immunobeads.

### Characterization of BEVs

The concentrations and size distributions of the BEVs resuspended in PBS were quantitatively assessed using a Nanoparticle Tracking Analyzer (NTA; NanoSight NS300 system, Malvern Panalytical Ltd., Malvern, U.K.). This analysis was conducted using NTA 3.1 software (Malvern Panalytical Ltd.), following the manufacturer's guidelines. The camera within the NTA system was finely adjusted to ensure that only particles emitting a distinct signal representing BEVs were measured, thereby ensuring accurate quantification and size distribution analysis of the vesicles. The percentage of BEVs in total EV population was calculated as follows: BEV% = [(EV_before_ - EV_after_) / EV_before_] × 100, where EV_before_ is the concentration of EV in patient's plasma before immunobeads addition and EV_after_ is the concentration of residual EV in patient's plasma after immunobeads addition.

### Analysis of GEO databases

To explore the miRNAs implicated in the onset and progression of BC, we performed extensive differential expression analysis of miRNAs utilizing 10 datasets from the National Center for Biotechnology Information (NCBI) GEO database (referenced in **Table S1**). Our analysis aimed to discern the patterns of miRNA expression in patients with BC from those in non-cancerous individuals, including those with benign tumors and healthy donors. This comparison involved the evaluation of clinical data derived from both tissues (datasets GSE26659, GSE44124, GSE45666, GSE97811, and GSE154255) and blood samples (datasets GSE42128, GSE73002, GSE98181, GSE110317, and GSE118782). Using volcano plots, we visualized the differential expression of miRNAs between patients with BC and controls, facilitating the identification of significantly upregulated or downregulated miRNAs in the context of breast cancer. Following the initial identification of DEMs, we further refined our analysis using Venn diagram comparisons. This step was performed using an online tool available at http://bioinformatics.psb.ugent.be/webtools/Venn/, which allowed the intersection of DEM lists from the aforementioned datasets.

### MiRNA profiles in BEVs

After isolating BEVs using the optimized method developed in this study, real-time polymerase chain reaction (PCR) was conducted to validate the miRNA profiles of BEVs. Briefly, miRNAs were extracted from BEVs using a Total Exosome RNA and Protein Isolation Kit (cat. 4478545; Thermo Fisher Scientific Inc.), according to the manufacturer's instructions. RNA concentration was measured using a Qubit™ microRNA Assay kit (cat. Q32880; Thermo Fisher Scientific Inc.) with a Qubit® 2.0 Fluorometer (cat. Q32866; Thermo Fisher Scientific Inc.). The extracted RNA was reverse-transcribed using a TaqMan microRNA Reverse Transcription Kit (cat. 4366597; Thermo Fisher Scientific Inc.). Candidate miRNAs, including miR-21-5p, miR-106b-5p, miR-155, miR-181a, miR-484, and miR-1290, were selected from public datasets. The differential expression levels of candidate miRNAs were measured by cDNA amplification using TaqMan Universal PCR Master Mix, No AmpErase UNG (cat. 4324018; Thermo Fisher Scientific Inc.), and TaqMan microRNA Assay kit (cat. 4440887; Thermo Fisher Scientific Inc.) on a CFX96 Real-time PCR system (cat. 3600037; Bio-Rad Laboratories Inc., Hercules, CA, USA). Individual miRNAs were reverse-transcribed with the following conditions: 30 min at 16 °C to anneal primers, 30 min at 42 °C for the extension, and 5 min at 85 °C to stop the reaction. Then, real-time PCR was run using cDNA with the following conditions: 10 min at 95 °C for enzyme activation, followed by 40 cycles consisting of denaturing at 95 °C for 15 s and annealing and elongation at 60 °C for 10 min. The miRNA expression levels were normalized using miR-16-5p as an internal control for exosomal miRNAs. All experiments were performed according to the manufacturer's instructions. The 2^-ΔΔCT^ method was used to determine the relative expression of miRNAs in BEVs.

### Assessment of reproducibility and repeatability of miRNA Ct values

To assess the reproducibility of miRNA Ct values, Bland-Altman plots were generated to visually evaluate the consistency of the Ct values for each miRNA in cancer patient samples (n = 120). For each miRNA, the differences between paired Ct values from repeated measurements were plotted against their averages. The mean difference (bias) was calculated, along with the 95% limits of agreement (LoA), defined as the mean difference ± 1.96 times the standard deviation of the differences. Furthermore, to assess the repeatability of the miRNA measurements, a time series plot was generated over a 5-day period across different sample types, including normal controls, benign patients, and cancer patients, with three samples per group. These measurements were conducted by a single experimenter to ensure consistency. All analyses were performed using GraphPad Prism to visually evaluate the reproducibility (**Figure S2**) and repeatability (**Figure S3**).

### ROC analysis

MedCalc software (v20.014; MedCalc Software Ltd., Ostend, Belgium) was used to conduct ROC and precision-recall curve analyses for each miRNA and miRNA signature. Univariate ROC analysis was utilized for each miRNA target to obtain the ROC curve, AUC, AUC standard error (SE), and 95% CI for evaluating the diagnostic power of the miRNA marker combinations. After performing a univariate ROC analysis on each combination of miRNA targets, the 'outstanding' combination with the highest AUC was selected, along with the lowest SE of AUC 24.

### MiRNA signature and mammography analysis

To assess the clinical feasibility of the miRNA signature, along with screening mammography, we classified all radiological data assessments, including mammography and ultrasound, using assessment codes from the American College of Radiology Breast Imaging Reporting and Data System (ACR BI-RADS) assessment category 25. BI-RADS employs a standard seven-point coding system ranging from 0 to 6 to describe the final work-up assessment. Assessments for each BI-RADS category for each breast were recorded as follows: 1, negative; 2, benign; 3, probably benign; 4a, low suspicion; 4b, intermediate suspicion; 4c, moderate suspicion; and 5, highly suggestive of malignancy. In our analysis, to minimize bias, we excluded BI-RADS category 6, which was reserved for patients already confirmed to have breast cancer through biopsy. Additionally, we designated BI-RADS category 0 (incomplete) as a screening examination that required subsequent imaging for a complete workup assessment. An incomplete initial assessment should be followed by further radiological workup, leading to the assignment of a workup assessment code from 1 to 5. Therefore, in situations where a positive screening result was obtained, BI-RADS category 0 was considered an incomplete situation where breast cancer could not be definitively diagnosed, and additional testing was necessary. Thus, it was categorized as 'Negative' for the purposes of this study. Additionally, we recorded the overall mammographic breast density according to the ACR BI-RADS assessment, categorized as A (almost entirely fatty, < 25%), B (scattered areas of fibroglandular density, 25 - 50%), C (heterogeneously dense breasts, 51 - 75%), or D (extremely dense breasts, > 75%).

### Statistical analysis

Each real-time PCR experiment was independently performed in duplicate. Data are presented as the mean ± standard deviation. All statistical analyses were performed using either an unpaired Student's *t*-test or multiple comparison tests following one-way analysis of variance (ANOVA) using Prism 10 (v10.2.0; GraphPad Software, San Diego, CA, USA). *p < 0.05; **p < 0.01; and ***p < 0.001 were used to indicate a statistically significant result, whereas “ns” represents non-significant results. In 10 public dataset analyses, differences in miRNA expression between the BC group and normal control group were compared using unpaired Student's *t*-test, and the criteria for statistical and clinical significance were adjusted *p*-value of < 0.05 and a fold change of ≥ 2. To identify the optimal miRNA signature for clinical performance, we compared the AUC values for each miRNA using a bivariate binomial model. Logistic regression analysis was conducted to elucidate the relationship between miRNAs and to treat them as independent variables. Logistic regression generates coefficients, along with the corresponding standard errors and significance levels, to develop a predictive formula for the logit transformation of the probability (Logit(p)) of the presence of the characteristic of interest. In this study, a dichotomous dependent variable was used as the predicted probability index. The miRNAs were included as independent variables in the logistic regression model. The significance level (α) was set to 0.05, and variables with p-values exceeding 0.1 were excluded from the model. Logistic regression coefficients were then computed to establish a dichotomous dependent variable for the influence of each miRNA on the outcomes. The goodness of fit of the logistic regression model was assessed using the overall model fit statistics. A p-value of less than 0.05 was considered indicative of at least one independent variable contributing significantly to the prediction of the outcome. Statistical analyses were performed using the MedCalc software (v20.014; The MedCalc Software Ltd., Ostend, Belgium). To identify optimal miRNA signatures, the AUC values of each probability index generated by logistic regression were compared.

## Results

### Optimization of immunoaffinity methods to isolate breast cancer-derived extracellular vesicles (BEVs)

**Figure 1:**
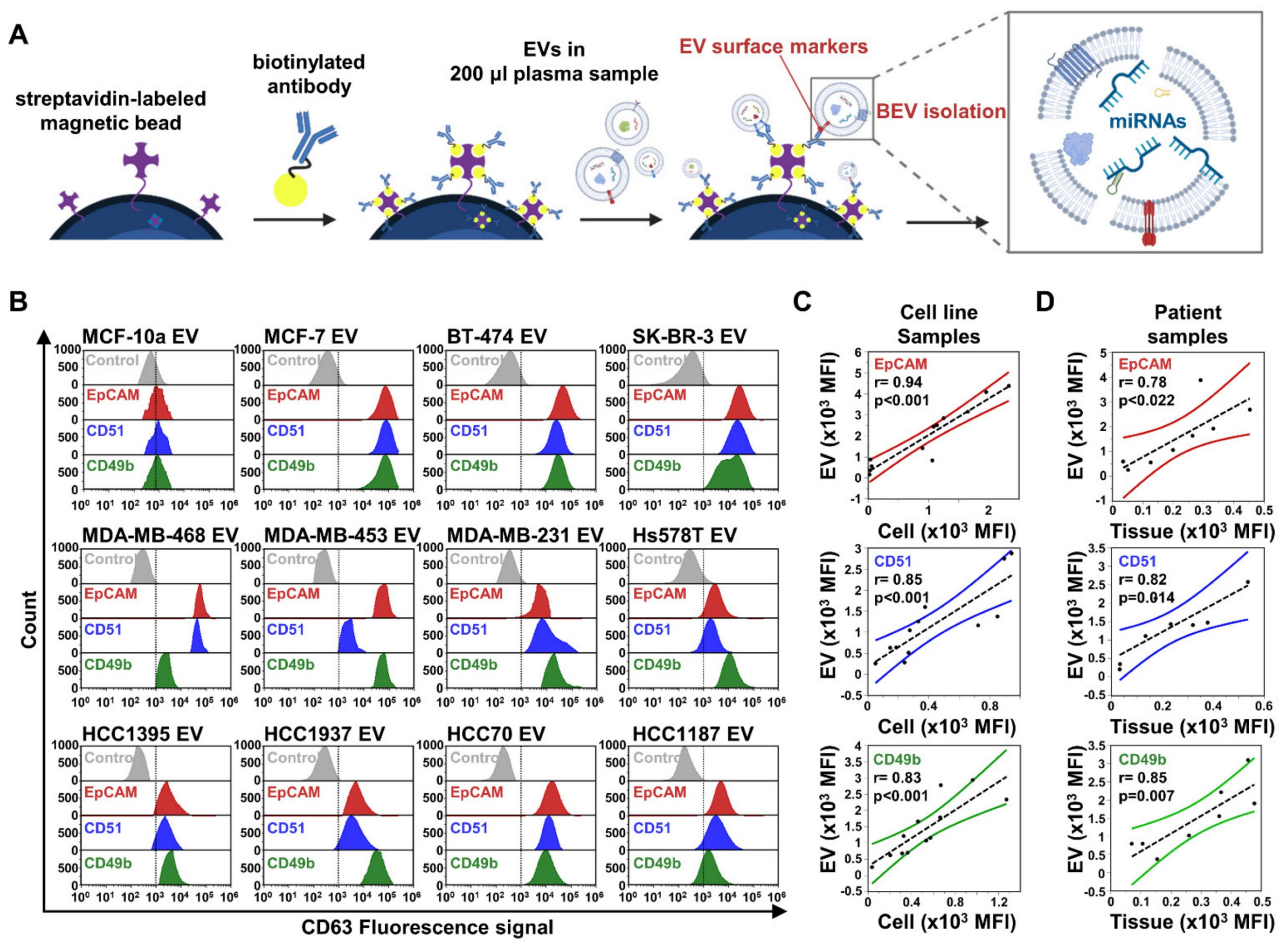
** Comprehensive analysis of breast cancer-derived extracellular vesicle (BEV) isolation and characterization.** (A) Schematic illustration of the extracellular vesicle isolation process using streptavidin-labeled magnetic beads coupled with biotinylated antibodies, targeting BEVs for subsequent miRNA analysis. (B) Flow cytometry analysis of BEVs isolated from 11 breast cancer cell lines and one control cell line (MCF-10a). EVs were labeled with CD63-PE-Cy7 as a general EV marker. Histograms show the fluorescence intensity for each marker—EpCAM (red), CD51 (blue), and CD49b (green)—across different cell lines. (C) Correlation analysis between EV and cell surface marker expression in 11 breast cancer cell lines for EpCAM (red), CD51 (blue), and CD49b (green). The Pearson correlation coefficient (r) and corresponding p-value indicate the significance of the correlations. (D) Correlation analysis extended to patient samples, including 5 cancer tissues and 3 benign tumor tissues, along with their paired plasma-derived EVs for EpCAM (red), CD51 (blue), and CD49b (green).

We focused on evaluating miRNA expression in BEVs toward early stage breast cancer diagnosis. Under the defined optimal conditions (**Figure S1**), we enriched BEVs using immunocapture separation and subsequently analyzed their miRNA expression (**Figure 1A**). To enhance the specificity and sensitivity of BEVs isolation, we focused on the enrichment of EVs relevant to a broad spectrum of subtypes, which enabled us to cover the intrinsic heterogeneity of breast cancer. We prepared magnetic beads conjugated with antibodies targeting specific breast cancer markers found in EVs, including epithelial cell adhesion molecules (EpCAM or CD326), CD49f, CD51, CD49b, and glypican-1 (GPC-1) (**Figure 1B**). Using these beads, we successfully isolated EVs from 11 breast cancer cell lines, observing a rightward shift in the fluorescence signal, indicating effective capture, whereas the control breast epithelial cell line MCF-10a showed negligible binding. While GPC-1 was effective in isolating EVs from the MCF7 and BT-474 cell lines, its overall performance was insufficient to consider it a primary marker for EV isolation (**Figure S4A**). In contrast, EpCAM demonstrated greater efficiency in capturing significant amounts of EVs from the MCF7, BT-474, and SK-BR-3 cell lines, which represent luminal and HER2 breast cancer subtypes with epithelial characteristics. However, its effectiveness was notably reduced in most triple-negative breast cancer (TNBC) cell lines.

Considering the tendency of TNBC to undergo epithelial-mesenchymal transition (EMT) — a process whereby epithelial cells gain mesenchymal and fibroblast-like properties, thus making tumor cells more motile, invasive, and prone to recurrence and metastasis — relying on a single marker is insufficient to cover all breast cancer subtypes 26. Therefore, we targeted CD49f, CD51, and CD49b (integrin family subunits) for TNBC EV isolation, in addition to EpCAM. Our findings revealed that EpCAM, CD51, and CD49b were prevalent in EVs from both non-TNBC (MCF-7, BT-474, and SK-BR-3) and TNBC (MDA-MB-468, MDA-MB-453, MDA-MB-231, Hs578T, HCC1395, HCC1937, HCC70, and HCC1187) cells. Notably, CD49f was effective only in targeting certain TNBC types, such as HCC1395, MDA-MB-231, and HCC70 (**Figure S4A**). However, CD51 and CD49b were abundantly present in EVs across all TNBC types, reflecting the diverse nature of TNBC; thus, we selected EpCAM, CD51, and CD49b as BEV isolation markers for potential clinical use.

To investigate the relationship between the expression of surface markers on cells and their corresponding EVs, Pearson's correlation analysis was performed across 11 breast cancer cell lines (**Figure 1C**). The analysis revealed strong correlation coefficients between the expression of surface markers on isolated EVs and their parent cells, with particularly high correlations observed for EpCAM (r = 0.94, ***p < 0.001), CD51 (r = 0.85, ***p < 0.001), and CD49b (r = 0.83, ***p < 0.001) 27. In contrast, CD49f (r = 0.59, *p < 0.05) and GPC-1 (r = 0.73, **p < 0.01) exhibited moderate to good correlations (**Figure S4B**). Furthermore, we extended the analysis to include 5 cancer tissues and 3 benign tumor tissues, along with their paired plasma-derived EVs, focusing on the expression of EpCAM, CD51, and CD49b. The results similarly revealed significant positive correlations between the expression levels of EpCAM (r = 0.78, p = 0.022), CD51 (r = 0.82, p = 0.014), and CD49b (r = 0.85, p = 0.007) in tumor tissues and those observed in paired EVs isolated from plasma samples (**Figure 1D**). These findings suggest that the selected markers—EpCAM, CD51, and CD49b—not only serve as reliable surface markers of tumor origin, reflecting cellular information in EVs with a strong positive association, but also demonstrate potential as consistent indicators of tumor-derived EVs in clinical samples, reinforcing their utility for diagnostic and monitoring purposes.

### Physicochemical property analysis of isolated BEVs

**Figure 2:**
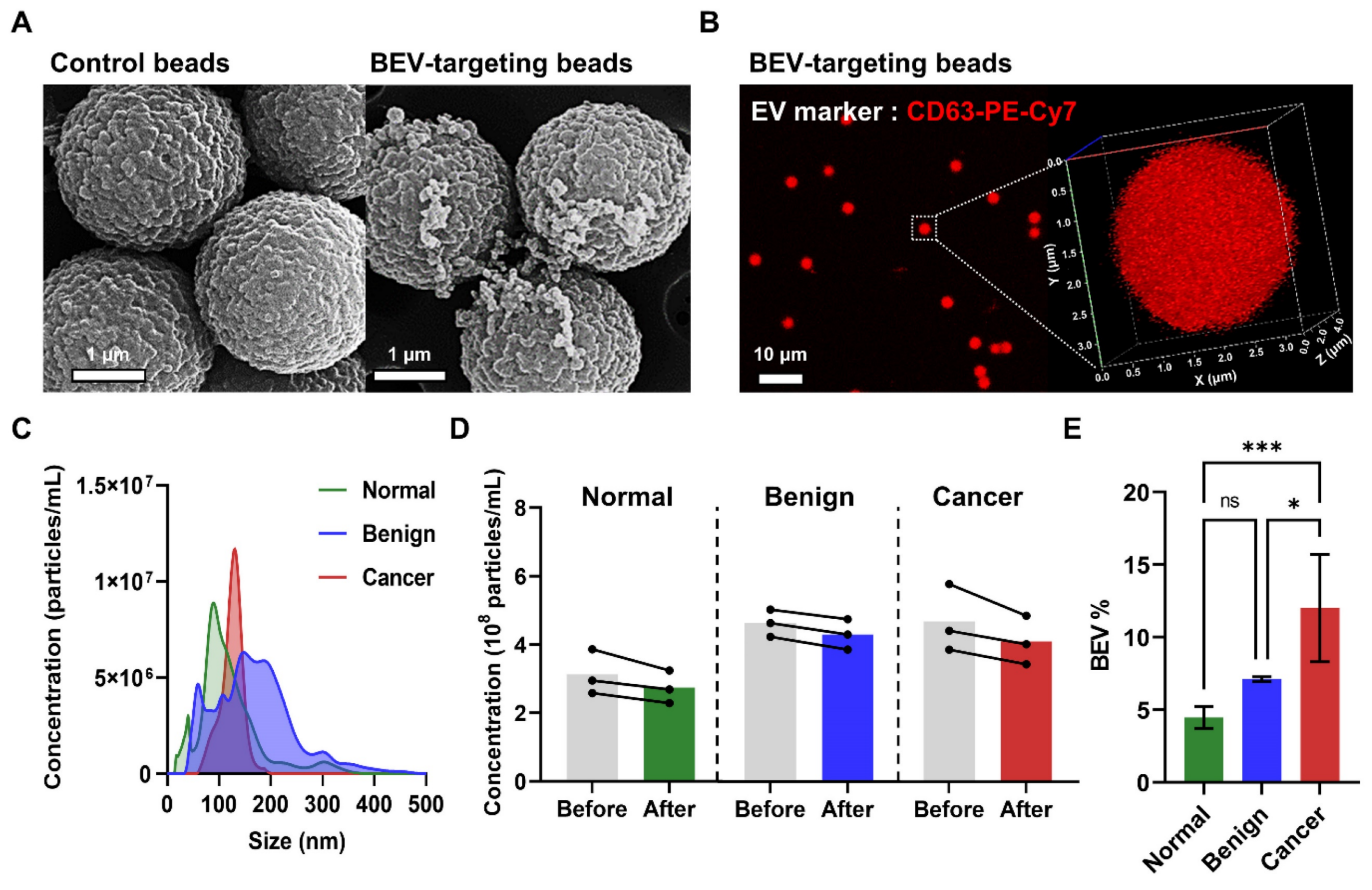
** Characterization and isolation efficiency of breast cancer-derived extracellular vesicles (BEVs).** (A) SEM images comparing control beads with BEV-targeting beads (coated with EpCAM, CD49b, and CD51 antibodies) after incubation with patient plasma samples. (B) Confocal microscopy image of BEV-targeting beads labeled with anti-CD63-PE-Cy7, a key EV marker. The 3D visualization confirms successful BEV capture, indicated by red fluorescence on the bead surface. (C) NTA showing the size distribution of EVs from normal (green), benign (blue), and cancer (red) groups. (D) Concentration of EVs before (gray) and after immunocapture across normal (green), benign (blue), and cancer (red) groups. (E) BEV isolation efficiency (BEV%) comparison among normal individuals, benign patients, and breast cancer patients. Statistical analysis was performed using one-way ANOVA; 'ns' indicates non-significant results; *p < 0.05, ***p < 0.001 indicate levels of significance.

SEM images of patient plasma samples revealed a significantly greater number of EVs attached to the surface of BEV-targeting beads (coated with EpCAM, CD49b, and CD51 antibodies) compared to control beads, demonstrating the effectiveness of the immunocapture process (**Figure 2A**). Confocal imaging and 3D bead visualization further confirmed the capture of BEVs by detecting red fluorescence signals corresponding to CD63, a key EV marker, on the bead surface (**Figure 2B**). These findings affirmed that the isolated vesicles exhibit characteristic EV markers, validating the immunocapture method.

Then, nanoparticle tracking analysis (NTA) was conducted to assess the yield and size distribution of the isolated EVs across samples from normal individuals, benign patients, and breast cancer patients. The isolated EVs across all groups were within the expected size range of 150 - 200 nm, consistent with BEVs (**Figure 2C**). Moreover, a reduction in the overall EV quantity before and after the immunocapture process was observed in all groups, confirming successful BEV isolation (**Figure 2D**). Notably, the BEV isolation efficiency (BEV%) varied among the groups, with efficiencies of 4.47% ± 0.75% for normal individuals, 7.12% ± 0.16% for benign patients, and 12.0% ± 3.7% for breast cancer patients (**Figure 2E**). This variation suggested a significantly higher presence of BEVs in the blood of breast cancer patients, supporting the hypothesis that these EVs are tumor-derived.

### Selection of candidate miRNAs in BEVs

**Figure 3:**
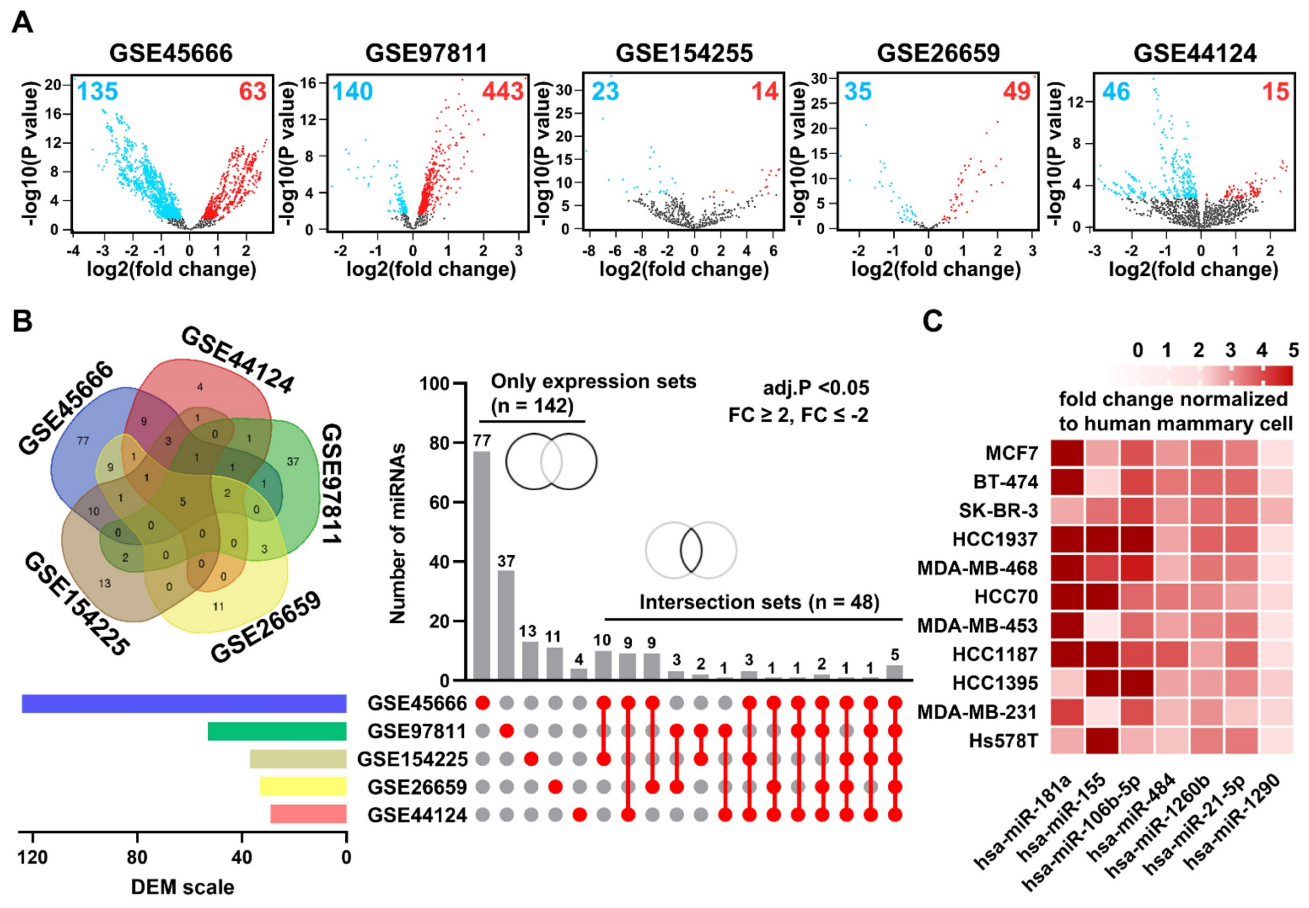
** Analysis of differentially expressed microRNAs (DEMs) in breast cancer.** (A) Volcano plots of DEMs in breast cancer tumor tissues compared to adjacent normal tissues from five public datasets: GSE45666, GSE97811, GSE154255, GSE26659, and GSE44124. Red and blue dots indicate microRNAs with high and low expression, respectively. (B) Venn diagram and Upset plot illustrating the distribution and overlap of DEMs among the five datasets. Horizontal bars show the total number of DEMs identified in each dataset, and vertical bars depict the number of DEMs common to multiple datasets, highlighting unique and intersecting sets. (C) Heatmap displaying the expression levels of candidate microRNAs enriched in breast cancer-derived extracellular vesicles (BEVs), isolated from cell culture media of various breast cancer cell lines, normalized against the MCF-10a control cell line. The heatmap specifically focuses on up-regulated DEMs identified from the comparison of the five GEO datasets selected for further analysis as potential biomarkers in BEVs.

The Gene Expression Omnibus (GEO) database was used to identify miRNA candidates for breast cancer diagnosis. We identified 379 downregulated and 584 upregulated differentially expressed miRNAs (DEMs) in tissue samples across five GEO datasets (**Figure 3A**). However, only 28 downregulated and 80 upregulated DEMS in blood samples were identified across additional 5 GEO datasets (**Figure S5**). When narrowed down by filtering with strict conditions (adjusted P-value < 0.05 and a fold change ≥ 2, ≤ -2), the contrast became even sharper (**Figure S6**). From our analysis, among 142 independent DEMs and 48 DEMs in common across 5 GEO datasets on breast cancer, we selected seven candidate miRNAs, namely miR-21, miR-106b, miR-181a, miR-484, miR-1260b, miR-155, and miR-1290, as likely to be significantly expressed in BEVs (**Figure 3B**). Subsequently, we measured the expression levels of these candidate miRNAs in BEVs from breast cancer cells and compared them with those in EVs from human mammary cells (MCF-10a) using our BEV isolation method (**Figure 3C**). The expression profiles of seven miRNAs were considerably elevated, supporting their potential as surrogate markers for breast cancer diagnosis.

**Figure 4:**
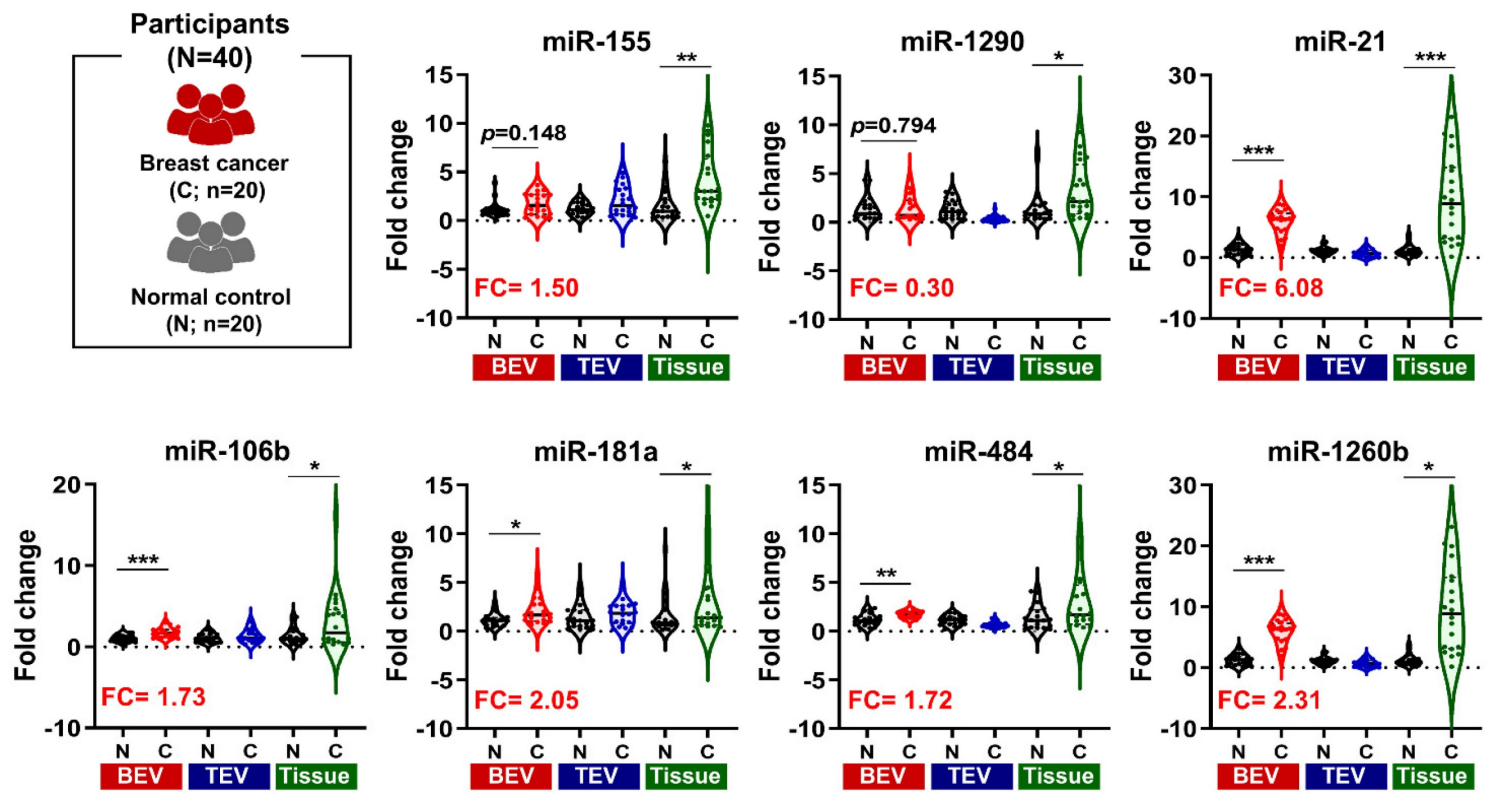
** Comparison of miRNA Expression in BEV, TEV, and Tissue.** Violin plots illustrating the gene expression levels of seven microRNAs (miRNAs). These levels are shown across tumor tissues, total extracellular vesicles (TEV), and breast cancer-derived extracellular vesicles (BEV) compared to their respective controls. The fold change (FC) of each miRNA is presented on the y-axis, while the x-axis categorizes the samples into normal (N) and cancer (C) groups for each sample type. Tissue samples were normalized to adjacent normal breast tissue, while plasma from healthy individuals served as controls for TEV and BEV samples. Statistical significance was assessed using unpaired Student's *t*-tests, with *p < 0.05, **p < 0.01, ***p < 0.001 indicating levels of significance. miRNAs with significant fold changes in BEV samples are highlighted in bold red letters.

To investigate the expression patterns of the seven targeted miRNAs, we analyzed three types of samples: tumor tissues, total extracellular vesicles (TEVs) isolated through EV precipitation, and BEVs isolated using an immunoaffinity approach (**Figure 4**). Our comparative analysis revealed that all seven miRNAs were markedly upregulated in the tumor tissues. In contrast, these miRNAs were not significantly upregulated in TEVs. Notably, five miRNAs in BEVs (miR-21, miR-106b, miR-181a, miR-484, and miR-1260b) were considerably elevated. Their expression levels in the breast cancer patient group were 6.08-, 1.73-, 2.05-, 1.72, and 2.31-fold higher than those in the normal control group, respectively.

**Figure 5:**
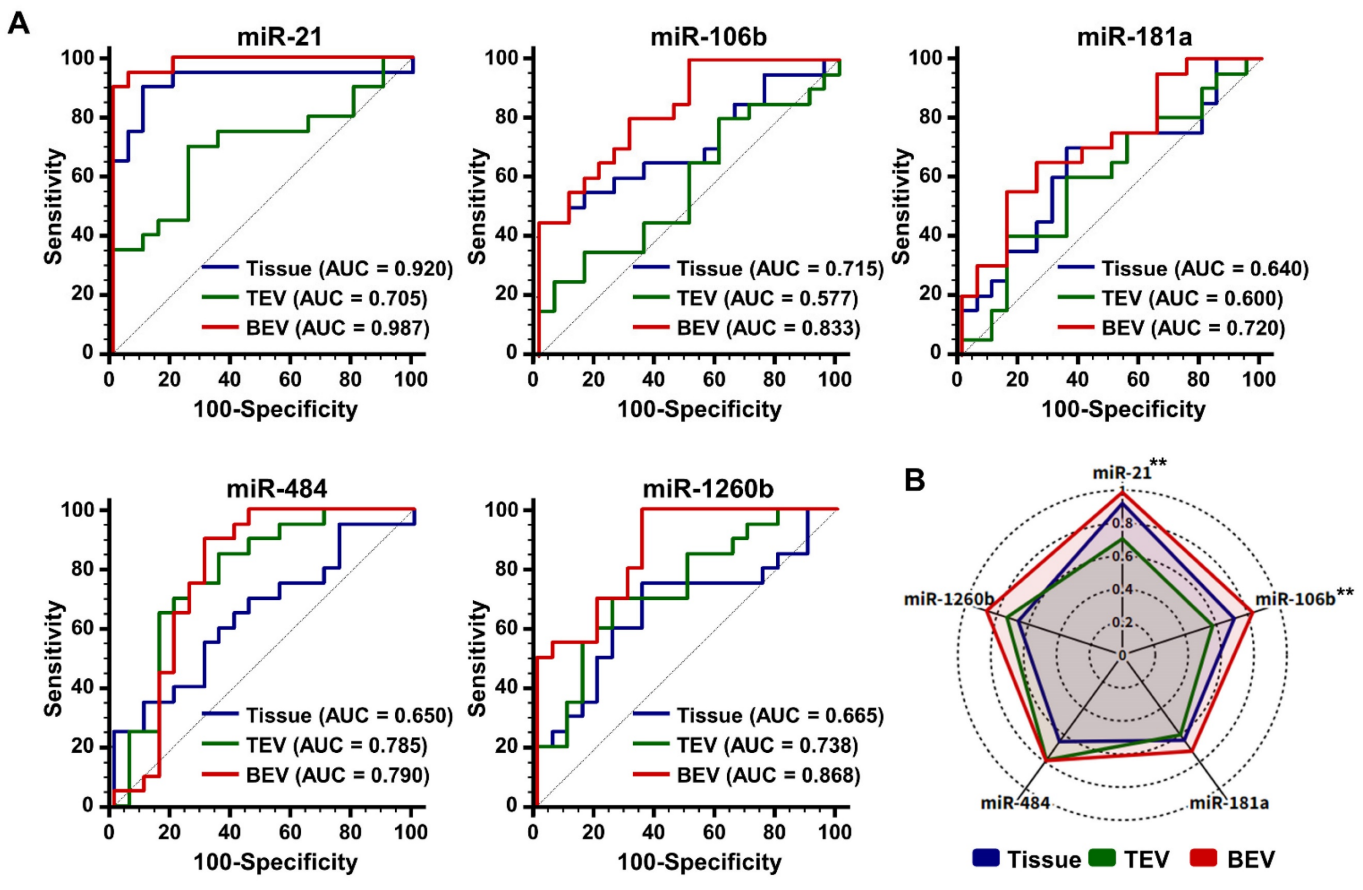
** Diagnostic performance of microRNAs (miRNAs) in breast cancer.** (A) Receiver operating characteristic curves illustrating the diagnostic capability of candidate miRNAs from tissue, total extracellular vesicles (TEVs), and breast cancer-derived extracellular vesicles (BEVs) in distinguishing 20 breast cancer patients from 20 healthy controls. Each curve represents a different miRNA (miR-21, miR-106b, miR-181a, miR-484, and miR-1260b), with corresponding area under the curve (AUC) values indicating their diagnostic accuracy. (B) A radar chart depicting the AUC values of each miRNA, demonstrating their potential effectiveness in breast cancer diagnosis. The chart compares the performance across three sample types: tissue, TEVs, and BEVs. Differences in AUC values between TEVs and BEVs are evaluated using an unpaired Student's *t*-test, with **p < 0.01 indicating statistically significant differences.

Given the increased expression of the five candidate miRNAs in breast cancer (BC) patients, we assessed their diagnostic potential using receiver operating characteristic (ROC) curve analysis (**Figure 5A**). This analysis revealed that the area under the curve (AUC) values for candidate miRNAs in BEVs, TEVs, and tumor tissues ranged from 0.790 to 0.987, 0.577 to 0.785, and 0.640 to 0.920, respectively. Surprisingly, the diagnostic performance of the candidate miRNAs in BEVs was superior, with all AUC values exceeding 0.7, which distinguished them from their performance in TEVs and tissue samples (**Figure 5B**). In particular, miR-21 and miR-106b showed significant differences in AUC values between TEV and BEV samples (**p < 0.01), highlighting their strong potential as diagnostic markers. These findings indicate that precise isolation of EVs from tumors allows miRNA analyses to more accurately reflect the molecular characteristics of the tumor. Accordingly, miR-21, miR-181a, miR-106b, miR-484, and miR-1260b in the BEVs were identified as reliable miRNA candidates for enhancing breast cancer diagnostic approaches, emphasizing the importance of further validation in a broader patient cohort within this study.

### Diagnostic potential of candidate miRNAs in BEVs

**Figure 6:**
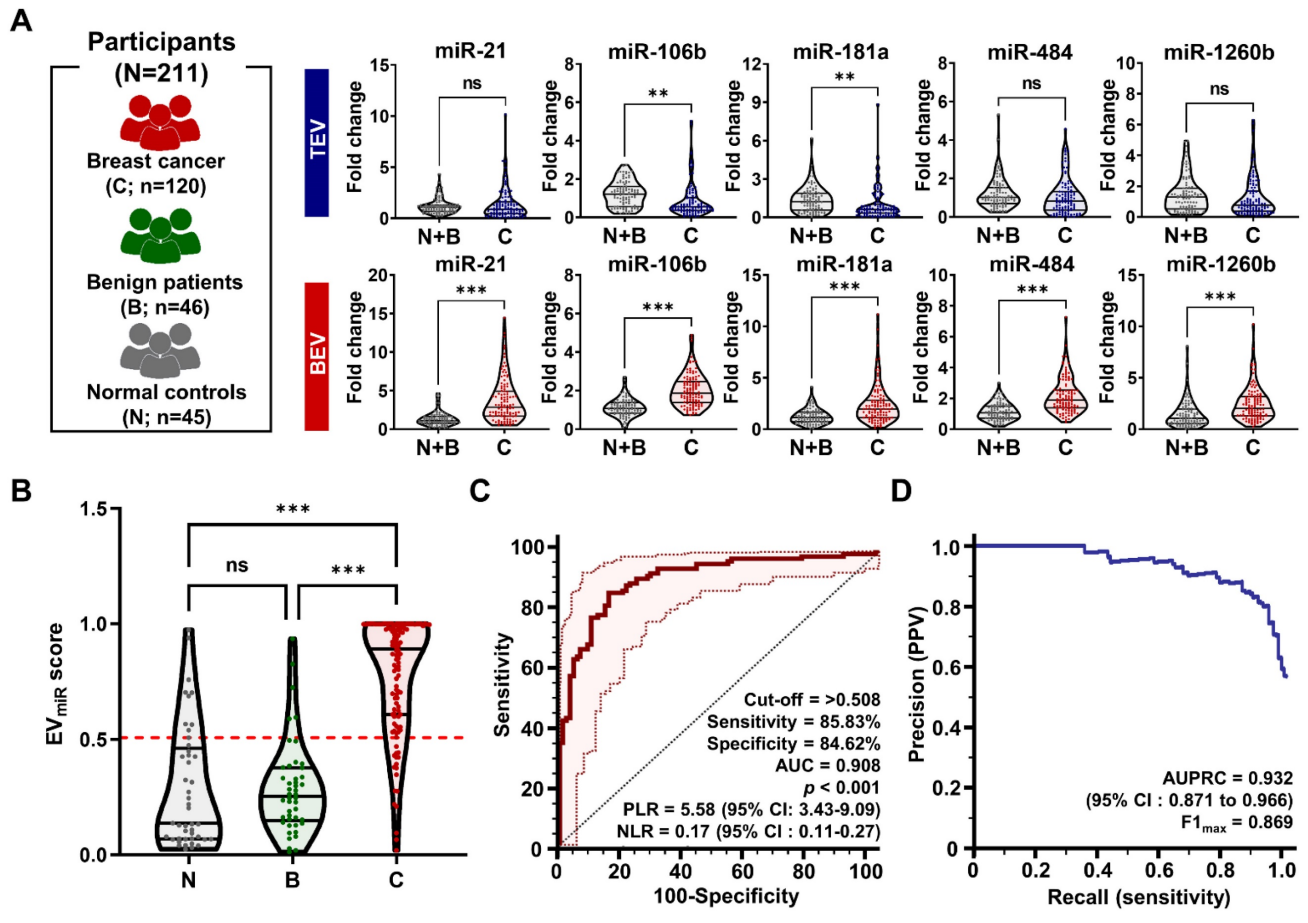
** Analysis of miRNA expression for breast cancer detection.** (A) Violin plots comparing the expression levels of selected microRNAs (miR-21, miR-106b, miR-181a, miR-484, and miR-1260b). These comparisons are made in TEVs and BEVs across different participant groups. 'C' stands for patients with breast cancer, 'B' for patients with benign tumor, and 'N' for normal controls. The plots highlight significant differences in miRNA expression between these groups. Statistical significance was assessed using unpaired Student's *t*-tests; 'ns' indicates non-significant results; *p < 0.05, **p < 0.01, and ***p < 0.001 indicate levels of significance. (B) A violin plot depicting the distribution of EV_miR_ scores across the groups. Scores for patients with breast cancer are compared against a combined control group consisting of individuals with benign tumors and normal controls. Statistical analysis was performed using a one-way ANOVA with Dunnett's multiple comparisons test; 'ns' indicates non-significant results, and '***p < 0.001' marks statistically significant differences. (C) Receiver operating characteristic (ROC) curve analysis assessing the sensitivity and specificity of the EV_miR_ score in distinguishing patients with breast cancer from the overall control group. The analysis details the cutoff value, sensitivity, specificity, positive likelihood ratio (PLR), negative likelihood ratio (NLR), and area under the ROC curve (AUC). (D) Precision-recall curve analysis to evaluate the precision and recall of the EV_miR_ score in identifying patients with breast cancer, highlighting the area under the precision-recall curve (AUPRC) and confidence intervals (CI).

A comparative analysis was performed between BEV-derived and TEV-derived miRNAs in 211 participants to assess whether the candidate miRNAs could act as potential early diagnostic biomarkers in breast cancer. The larger cohort included 120 patients with breast cancer, 46 patients with benign breast disease, and 45 healthy controls. As indicated in **Figure 6A**, miR-21, miR-484, and miR-1260b in TEVs did not display statistically significant differences between patients with breast cancer and control groups (benign and healthy controls), whereas miR-106b and miR-181a were expressed at lower levels. Remarkably, all miRNA candidates were significantly upregulated in BEVs (***p < 0.001) than in TEVs. A detailed logistic regression analysis of the 26 miRNA combinations was performed to optimize the clinical performance of the top five miRNAs in BEVs (**Table S2**). The selection of the optimal miRNA signature was primarily influenced by a high AUC value (greater than 0.9) and notable differences in performance between TEVs and BEVs. This approach ensures that the chosen miRNA signature not only meets the high standards of diagnostic accuracy but also reflects distinct tumor information. Combination 26, which comprised miR-21, miR-106b, miR-181a, miR-484, and miR-1260b, was the most effective and thus chosen as the miRNA signature for potential clinical use (**Table 1**). This signature was subsequently used to compute the predictive EV_miR_ scores for further evaluation.

Through a combination analysis, we screened the EV_miR_ scores among patients with benign tumors, patients with malignant tumors, and healthy volunteers (**Figure 6B**). The score was considerably higher in patients with breast cancer than in those with benign breast disease and normal controls, with no significant differences noted between the benign and normal groups. This indicates that the EV_miR_ score can distinguish breast cancer from benign or normal conditions. Further ROC analysis assessed the clinical performance of the score in diagnosing breast cancer (**Figure 6C**), revealing a sensitivity of 85.83% (95% confidence interval [CI]: 78.3 - 91.5%) and a specificity of 84.62% (95% CI: 75.5 - 91.5%) at a threshold of 0.508. The AUC was 0.908 (95% CI: 0.861 - 0.943), indicating the strong diagnostic capability of the miRNA signature. Notably, the positive likelihood ratio (PLR) of 5.58 (95% CI: 3.43 - 9.09) suggests that a positive test result makes the condition 5.58 times more likely, while the negative likelihood ratio (NLR) of 0.17 (95% CI: 0.11 - 0.27) indicates a lower probability of the condition following a negative test result. These ratios provide critical insights into the diagnostic precision of the test (**Table S3**). However, clinical performance metrics could be influenced by sample selection bias within the controlled study population, potentially leading to imbalances. To mitigate this and support diagnostic validity for breast cancer, the area under the precision-recall curve (AUPRC) and maximum F1 score (F1_max_) were calculated (**Figure 6D**). The AUPRC and F1max values were 0.932 and 0.869, respectively, demonstrating the efficacy of the method in predicting positive classes despite class imbalances.

### The evaluation of predictive EV_miR_ score in patients with BC based on disease status

To reduce confounding bias, further analysis was performed to explore the relationship between the EV_miR_ score and various clinical characteristics of patients with breast cancer categorized by disease status (**Table 2**).

**Figure 7:**
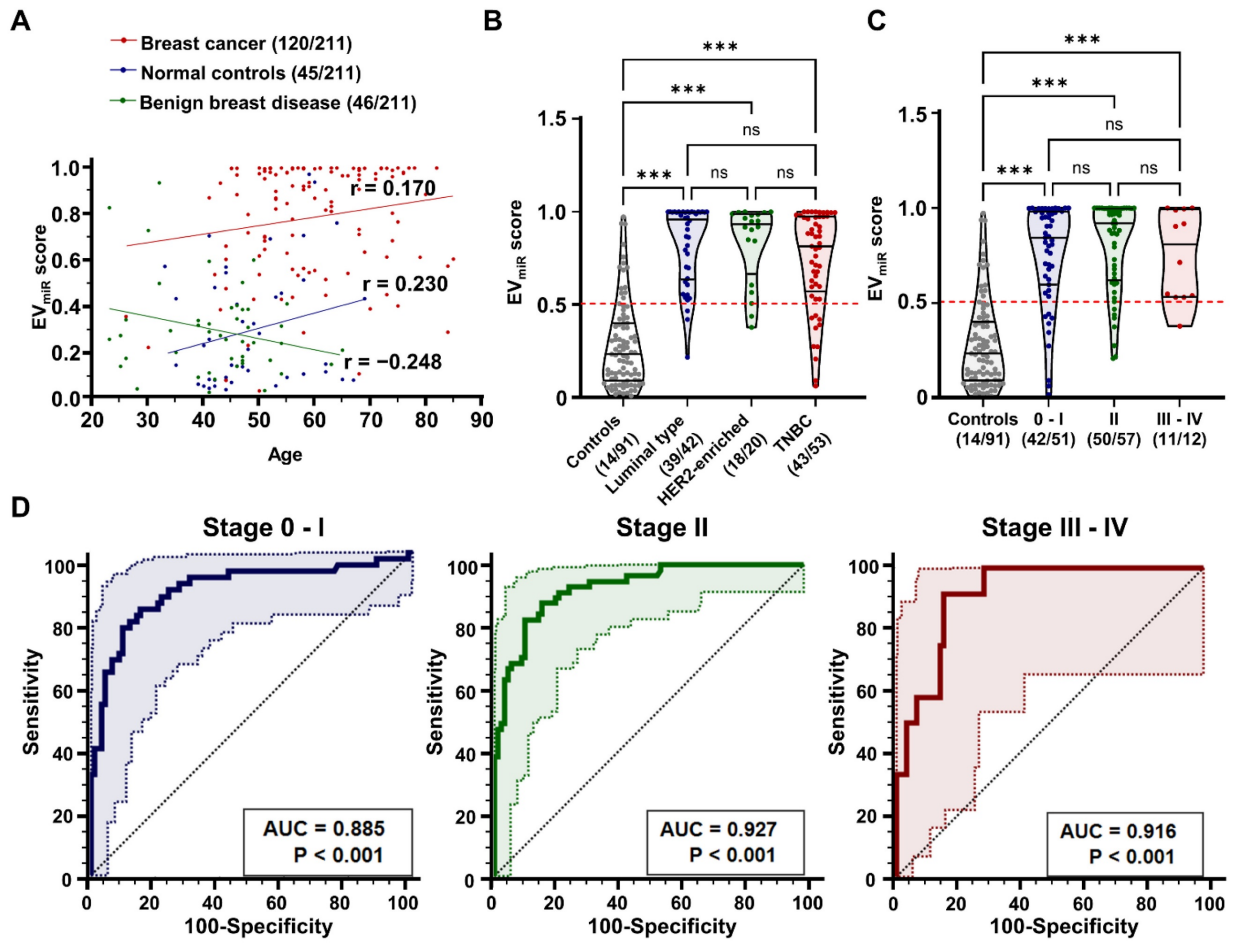
** Analysis of EV_miR_ score's clinical correlations and diagnostic performance in breast cancer.** (A) Scatter plot demonstrating the correlation between age and the EV_miR_ score among different participant groups: patients with breast cancer, normal controls, and patients with benign breast disease. The Pearson correlation coefficient (r) values are shown for each group, indicating varying degrees of correlation between age and the EV_miR_ scores. (B) Violin plots comparing the EV_miR_ scores across molecular subtypes of breast cancer, including luminal, HER2-enriched, and triple-negative. Breast cancer cases with unclassified molecular subtypes (N/A) were excluded from the analysis (n = 5). The plots highlight significant differences in the scores with 'ns' indicating non-significant results and '***p < 0.001' indicating statistically significant differences, assessed using one-way ANOVA with Dunnett's multiple comparisons test. (C) Violin plots evaluating the EV_miR_ scores across different stages of breast cancer progression, categorized as Stage 0-I, II, and III-IV. The scores are compared against a control group of non-cancer participants, with statistical analysis shown as 'ns' for non-significant and '***p < 0.001' for statistically significant differences. (D) Receiver operating characteristic (ROC) curve analysis of the EV_miR_ score's diagnostic performance according to TNM staging of breast cancer. Significance levels (***p < 0.001) are noted below the area under the ROC curve (AUC) values.

Clinical variables such as tumor size (*p* = 0.297), lymph node invasion (*p* = 0.215), distant metastasis (*p* = 0.751), ki-67 expression (*p* = 0.345), recurrence (*p* = 0.234), and survival (*p* = 0.356) were not significantly correlated with the EV_miR_ score. Moreover, regarding key clinical parameters such as age, subtype, and stage, our results indicated a low correlation between the EV_miR_ score and age distributions within each participant group, with correlation coefficients of 0.170, 0.230, and -0.248, respectively (**Figure 7A**). Upon assessing the association with breast cancer subtypes, divided into luminal, HER2, and TNBC subtypes based on molecular biological characteristics, we found that the EV_miR_ score was consistently higher across all subtypes than in normal controls (**Figure 7B**). Additionally, there was no notable link between tumor progression from early to advanced stages (**Figure 7C**). ROC analysis for each stage yielded AUC values of 0.885, 0.927, and 0.916 (***p < 0.001), respectively, indicating excellent diagnostic performance across stages but minimal differences depending on the stage (**Figure 7D**). These findings suggest that the EV_miR_ score is an effective diagnostic tool for breast cancer, regardless of age, subtype, or stage, highlighting its clinical value in early stage cancer detection.

### The potential utility of miRNA signature to compensate or complement mammography

**Figure 8:**
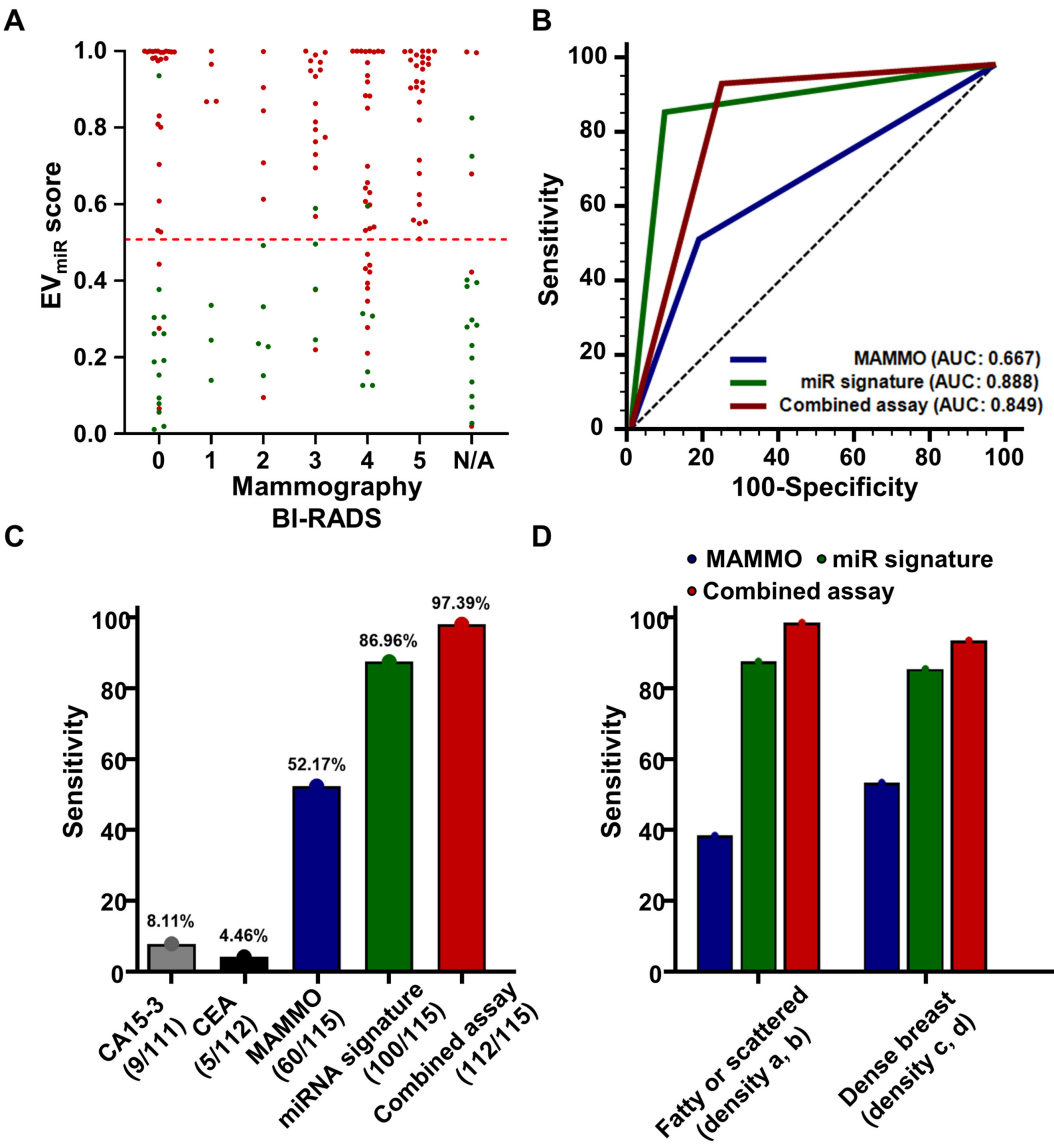
** Diagnostic performance of EV_miR_ score and comparisons with conventional diagnostic methods.** (A) Scatter plot depicting the EV_miR_ scores against BI-RADS categories used in mammography, ranging from 0 to 5, with N/A representing non-assessable categories. Each point represents the EV_miR_ score for an individual patient. (B) Receiver operating characteristic (ROC) curves comparing the sensitivity and specificity of the EV_miR_ score against conventional mammography (MAMMO) and a combined assessment using both methods. The curves illustrate the area under the ROC curve (AUC) for each method, highlighting their diagnostic accuracy. (C) Bar graph showing the sensitivity of the EV_miR_ score in identifying breast cancer compared to the established cancer markers CA 15-3 and CEA, alongside a combined testing approach. Percentages above each bar indicate the sensitivity of each test. (D) Sensitivity of the EV_miR_ score stratified by breast density categories, comparing fatty or scattered density (categories a and b) versus dense breast tissue (categories c and d). The bars represent the sensitivity of the EV_miR_ score, the MAMMO, and the combined assay for each density category.

Mammography, a commonly used breast cancer screening method, often poses challenges in terms of clinical sensitivity, particularly because of dense breast tissue, leading to notable false negatives and the necessity for further diagnostic procedures 4. To address these limitations, we examined the distribution of miRNA signatures based on mammography findings in 115 breast cancers and 32 benign breast diseases with confirmed mammography results. Specifically, BI-RADS 0 denotes an "incomplete" classification, suggesting the need for additional imaging rather than an immediate tissue biopsy. Therefore, to assess the potential complementarity of the miRNA signature for BI-RADS 0 cases, we verified the true-positive and true-negative rates. Among the 40 patients initially classified as BI-RADS 0, comprising 27 with breast cancer and 14 with benign breast disease, miRNA signature analysis identified 24 patients with breast cancer (88.89%) as true positives and 13 benign cases (92.86%) as true negatives (**Figure 8A, Table S4**). Next, we assessed the clinical performance of mammography, miRNA signatures, and combined analysis of 115 patients with breast cancer and 32 patients with benign breast disease with confirmed mammography results. The mammography criteria defined BI-RADS 4 and 5 as positive, whereas in the combined analysis, a positive result in either test was considered an overall positive result. Our analysis yielded AUC values of 0.667 (95% CI: 0.585 - 0.743) for mammography, 0.888 (95% CI: 0.825 - 0.934) for the miRNA signature, and 0.849 (95% CI: 0.781 - 0.903) for the combined analysis (**Figure 8B**). Detailed analysis of clinical sensitivity, including CA15-3 and CEA, revealed sensitivities of less than 10% for these markers in breast cancer, whereas mammography demonstrated a sensitivity of 52.17% (**Figure 8C**). The miRNA signature exhibited a sensitivity of 86.96%, and the combined analysis showed a sensitivity of 97.39%, highlighting its potential utility in breast cancer diagnosis. Furthermore, we compared the clinical sensitivities of mammography, miRNA signature analysis, and combined analysis of dense breasts in 116 patients with breast cancer using retrospectively collected breast density information. The results indicated differences in sensitivity across various breast density categories. In 98 patients with breast cancer and dense breasts, mammography achieved a sensitivity of 54.08%, miRNA signature analysis achieved 86.73%, and combined analysis reached 94.90% (**Figure 8D, Table S5**). These findings underscore the potential of combining assays with miRNA signature analysis to enhance the diagnostic sensitivity of mammography, particularly in cases of dense breast tissue where mammography alone may exhibit lower sensitivity.

## Discussion

Development of blood tests that accurately reflect specific tumor characteristics using circulating biomarkers remains a significant challenge. Despite technological advances that have improved detection limits, overcoming the quantitative deficiencies inherent in circulating tumor DNA (ctDNA) and circulating tumor cells (CTC), especially in early-stage cancers, continues to pose difficulties 28, 29. Concurrently, EVs, which can be used to obtain molecular information from living tumor cells at an early stage, have emerged as a promising avenue for cancer diagnostics using liquid biopsy 30, 31. Unlike other circulating biomarkers, EV cargo is enriched and protected by a lipid bilayer structure, rendering it relatively unaffected by stability issues and background noise, thereby enhancing its utility for diagnostic purposes 32. While previous studies have often focused on surface proteins, such as tetraspanins (CD9, CD81, and CD63 among others), which are commonly overexpressed in EVs 33, our research aimed to isolate EVs that accurately reflect the unique characteristics of breast cancer, thereby complementing existing diagnostic methods and improving clinical performance relative to bulked TEVs and tissue-based approaches. We evaluated several EV surface proteins that are overexpressed across various molecular subtypes of breast cancer to demonstrate their broad applicability. By employing immunoaffinity technology, we successfully isolated BEVs, which exhibited superior clinical performance in miRNA analysis compared to tissue-derived samples and TEVs.

Despite analyzing large-scale public data with over thousand patient records, we observed significant discrepancy between the miRNA profiles in the blood and tissues, which highlighted several important insights for identifying accurate circulating miRNA markers for effective breast cancer diagnosis (**Figure S7**). Our main considerations were as follows (1) the selective release of miRNAs into the bloodstream, involving only certain subsets from the extensive miRNA pool; (2) the susceptibility of circulating miRNAs in the blood to nuclease degradation, unless they are stabilized through complexes with specific proteins such as high-density lipoproteins, argonate2, or encapsulation within EVs; and (3) the role of tumor tissues in modulating their environment via autocrine and paracrine interactions through EVs, given their miRNAs' role in critical cancer processes such as cell proliferation, apoptosis evasion, angiogenesis, and metastasis. Overlooking these intricate biological factors could lead to the selection of incorrect biomarkers and misinterpretation of the data. Therefore, our study focused on miRNAs within BEVs that reflect as much information as possible from the tumor tissue.

In this study, we focused on isolating breast cancer-derived extracellular vesicles (BEVs) to reduce noise from non-cancerous EVs and enhance the specificity of our analysis. This was achieved by targeting EpCAM, CD49b, and CD51, which play crucial roles in cell adhesion, migration, metastasis, and signaling — key processes in cancer progression 34-37. EpCAM is commonly overexpressed in carcinomas, making it a valuable tumor marker. CD49b, as part of the integrin α2β1 complex, binds to ECM components like collagen to facilitate cell adhesion and migration, while CD51, which forms integrin complexes with various β subunits, is involved in adhesion, angiogenesis, and apoptosis. By selectively isolating BEVs using these markers, we were able to focus on the miRNA profiles within these cancer-specific EVs. The primary objective of this study was to identify miRNA candidates within BEVs and assess their clinical utility in diagnosing breast cancer, thereby minimizing the impact of non-specific EVs and improving diagnostic accuracy.

Additionally, we identified a signature of BEV-derived miRNAs, including miR-21, miR-106b, miR-181a, miR-484, and miR-1260b, which were upregulated in patients with BC compared to those with benign breast disease and healthy individuals. These miRNAs play crucial roles in breast cancer progression by targeting and regulating key genes and pathways in tumor cells. For example, miR-21 facilitates breast cancer progression and metastasis by inhibiting tumor suppressor genes such as *PTEN* and *PDCD4*, which in turn activate the PI3K/AKT and MEK/ERK signaling pathways 38-41. Similarly, miR-106b accelerates breast cancer progression by targeting *PTEN*, boosting cell proliferation, migration, and invasion 42, 43. It also activates the Rho/ROCK1 pathway, which contributes to tumor growth 44. MiR-484, by targeting KLF4, increases the sensitivity of tamoxifen-resistant breast cancer cells and is commonly involved in critical signaling pathways related to breast cancer progression, indicating its potential as a diagnostic biomarker because of its high levels in the plasma and serum of patients with breast cancer 45-47. MiR-1260b is linked to more aggressive breast cancer characteristics, such as larger tumor size, advanced stage, lymph node invasion, and reduced overall survival 48, 49. It targets CASP8, adding to its potential as a biomarker, and enhances migration, invasion, and immune evasion by controlling CCDC134 and activating the MAPK signaling pathway 50.

Recent studies on the expression levels of these miRNAs in the blood have reported contradictory results. For example, some researchers have observed lower levels of miRNAs in the blood of patients with breast cancer, whereas others have reported elevated levels 51-56. These discrepancies highlight the complexity of miRNA regulation and suggest that their roles may differ depending on the specific conditions of the tumor environment. A review by Yang et al. emphasized conflicting findings regarding miR-181a-5p, suggesting that it acts as a dual regulator in different contexts 57. Another possible hypothesis is that the conventional methods used for analyzing miRNAs in the blood may not adequately reflect the actual state of tumor tissues. BEVs encapsulate miRNAs that are crucial for tumor communication, whereas TEVs may carry a different set of bulk miRNAs that reflect a broader physiological state. Indeed, our findings revealed considerable differences in miR-181a-5p expression between TEVs and BEVs, supporting its reliability in accurately identifying onco-miRNAs associated with breast cancer. Finally, the production and degradation rates of miRNAs can vary significantly over time, contributing to discrepancies in their profiles observed in different studies. Such variability can be influenced by factors such as disease stage, the patient's physiological state, and changes in the tumor environment. These dynamics emphasize the importance of conducting longitudinal studies to profile miRNAs in the same patient over multiple time points 58-60.

In addition to analyzing the clinical performance of the miRNA signature through BEV isolation, we reviewed its clinical utility to establish an intended use that could benefit existing breast cancer diagnostic procedures for clinical application. Mammography has high clinical specificity for breast cancer, making it a useful tool for screening purposes 61, 62. However, false negatives tend to occur owing to dense breasts, creating a bottleneck in the breast cancer diagnosis and evaluation process and posing a risk of developing interval cancer 2. We aimed to determine the potential and clinical utility of the validated miRNA signatures in BEVs for complementing imaging findings. Our aim was not to advocate for the interpretation of the combined assay in a breast screening setting or provide guidance on assigning subsequent diagnoses. Dealing with inconsistencies between radiological assessments and miRNAs poses a challenge, as there is limited precedent for such situations. In particular, it was a very cautious approach to compare and analyze clinical performance by setting 'Positive' in the BI-RADS 0 category. However, in our approach, we prioritized definite 'Positive' results in the positive screening assessment. A definite 'Positive' result indicated that further diagnosis was required. This decision was based on a clinical scenario in which any positive result would necessitate further diagnostic procedures. Therefore, the combined mammography and miRNA analysis was deemed 'Positive' if either result indicated a positive finding. We confirmed that the use of miRNA signatures in combination with mammography can compensate for the low clinical sensitivity of mammography in dense breasts. Our study validated the clinical performance of BEV-derived miRNA signatures, demonstrated their potential utility in breast cancer diagnosis, and highlighted avenues for future research and clinical applications.

However, to substantiate the 5- BEVs miRNA signature as a breast cancer biomarker, the following in-depth discussion is necessary. First, the specificity of the miRNA signature expression in breast cancer needs to be addressed. It is essential to determine whether the miRNA signature increases solely because of breast cancer. To achieve this, the demographic factors analyzed were limited to age, but considerations such as menopausal status, BMI, smoking, and alcohol consumption should be included, and whether these demographic factors contribute to the elevation of miRNA signatures in patients with breast cancer needs to be investigated. Second, there was bias in the distribution of the study population. The recruited patients with breast cancer were distributed across specific TNM stages, histological classifications, and molecular subtypes. This diverse distribution could lead to interpretative biases in the results, necessitating a cautious interpretation of the results. Third, there are limitations to the hypothesis that encompasses molecular subtypes. This study aimed to reflect tumor heterogeneity, which was not accounted for in previous studies that reported low sensitivity in breast cancer. To reflect breast cancer heterogeneity, molecular subtypes were comprehensively included by introducing EpCAM, CD49b, and CD51 into the immunoaffinity for BEV separation. Although this selective separation exhibited higher clinical performance than TEV separation diagnostic studies, we were not able to conclude that all breast cancer-specific EVs could be captured, and the possibility of missing breast cancer-specific EVs cannot be excluded.

## Conclusions

In this study, we verified the clinical performance of a miRNA signature obtained through the isolation of BEVs and reviewed its clinical utility. Additionally, we confirmed its potential for use in clinical practice. To the best of our knowledge, this clinical performance, specified for a combination of single analyte types, is the best reported clinical diagnostic performance. Our study results suggest that selectively separating EVs reflecting breast cancer heterogeneity to analyze the miRNA signature of BEVs provides an opportunity for the enrichment of tumor-derived EVs among heterogeneous EV populations in the blood. This allows for a multicomponent diagnostic window that utilizes nucleic acids, lipids, metabolites, and proteomes within the BEVs. In summary, our study underscores the enhanced clinical performance achieved by the isolation and analysis of tumor-derived EVs, highlighting the potential for introducing a more tumor-specific liquid biopsy approach into clinical practice.

## Supplementary Material

Supplementary figures and tables.

## Figures and Tables

**Table 1:** Comparison of the top five miRNA combinations

AUC (95% Confidential Interval)					
Rank	Variable	TEVs	BEVs	Difference	Remark
**1**	**Combination 26** **(miR-21, miR-106b, miR-181a, miR-484, miR-1260b)**	**0.680** **(0.612 - 0.744)**	**0.905** **(0.856 - 0.941)**	**33.09%**	**EV_miR_ score**
2	Combination 23 (miR-21, miR-106b, miR-484, miR-1260b)	0.682 (0.614 - 0.745)	0.906 (0.857 - 0.942)	32.84%	
3	Combination 21 (miR-21, miR-106b, miR-181a, miR-484)	0.682 (0.613 - 0.745)	0.892 (0.841 - 0.931)	30.79%	
4	Combination 12 (miR-21, miR-106b, miR-484)	0.684 (0.615 - 0.747)	0.893 (0.842 - 0.931)	30.56%	
5	Combination 1 (miR-21, miR-106b)	0.703 (0.635 - 0.764)	0.881 (0.828 - 0.922)	25.32%	

AUC, area under the curve; TEVs, total extracellular vesicles; BEVs, breast cancer-derived extracellular vesicles.

**Table 2:** Clinical characteristics of the enrolled patients with breast cancer

Variables	N (%)	Mean EV_miR_ score	95% CI	*p* value
**T stage**				
Tis (carcinoma in situ)	5 (4.17)	0.597	0.087 - 1.11	0.297
T1 (≤ 2 cm)	64 (53.33)	0.793	0.730 - 0.856	
T2 (> 2 cm, ≤ 5 cm)	44 (36.67)	0.788	0.717 - 0.858	
T3 (> 5 cm)	5 (4.17)	0.696	0.470 - 0.921	
T4 (grown into the chest wall or the skin)	2 (1.67)	0.993	0.955 - 1.03	
**N stage**				
N0 (none)	83 (69.17)	0.769	0.714 - 0.825	0.215
N1 (1 - 3 lymph nodes)	26 (21.67)	0.834	0.738 - 0.931	
N2 (4 - 9 lymph nodes)	3 (2.50)	0.971	0.857 - 1.086	
N3 (≥ 10 lymph nodes)	8 (6.67)	0.678	0.473 - 0.883	
**M stage**				0.751
M0	118 (98.33)	0.783	0.795 - 0.903	
M1	2 (1.67)	0.727	-1.529 - 2.982	
**ki-67**				0.345
Low (< 15%)	33 (30.00	0.806	0.724 - 0.888	
High (≥ 15%)	77 (70.00)	0.792	0.738 - 0.846	
**Recurrence**				
Yes	44 (36.67)	0.841	0.779 - 0.903	0.234
No	76 (63.33)	0.748	0.687 - 0.809	
**Survival**				
Death	19 (15.83)	0.842	0.743 - 0.941	0.356
Survive	101 (84.17)	0.771	0.721 - 0.822	

* Patients without test results were excluded from the analysis. CI, confidence interval.

## Abbreviations

WHO: World Health Organization
BEVs: Breast Cancer-derived Extracellular Vesicles
miRNA: MicroRNA
EV: Extracellular Vesicle
miRNA: MicroRNA
EpCAM: Epithelial Cell Adhesion Molecule
GPC-1: Glypican-1
TNBC: Triple-Negative Breast Cancer
EMT: Epithelial-Mesenchymal Transition
SEM: Scanning Electron Microscopy
NTA: Nanoparticle Tracking Analysis
GEO: Gene Expression Omnibus
DEMs: Differentially Expressed miRNAs
BC: Breast Cancer
ROC: Receiver Operating Characteristic
AUC: Area Under the Curve
PLR: Positive Likelihood Ratio
NLR: Negative Likelihood Ratio
AUPRC: Area Under the Precision-Recall Curve
F1max: Maximum F1 Score
BI-RADS: Breast Imaging Reporting and Data System
EMR: Electronic Medical Records
EDTA: Ethylenediaminetetraacetic Acid
PBS: Phosphate-Buffered Saline
RPMI-1640: Roswell Park Memorial Institute-1640 Medium
FBS: Fetal Bovine Serum
Mg bead: Magnetic Bead
Ab-to-Mg bead: Antibody-to-magnetic bead
FACS: Fluorescence-Activated Cell Sorting
PE-Cy7: Phycoerythrin-Cyanine 7
CD63: Cluster of Differentiation 63
OsO4: Osmium Tetroxide
NCBI: National Center for Biotechnology Information
PCR: Polymerase Chain Reaction
SE: Standard Error
CI: Confidence Interval
ACR: American College of Radiology
ANOVA: Analysis of Variance
ctDNA: Circulating Tumor DNA
CTC: Circulating Tumor Cells
TEV: Tumor-Derived Extracellular Vesicles
CD: Cluster of Differentiation
PTEN: Phosphatase and Tensin Homolog
PDCD4: Programmed Cell Death 4
PI3K/AKT: Phosphoinositide 3-Kinase/Protein Kinase B
MEK/ERK: Mitogen-Activated Protein Kinase/Extracellular Signal-Regulated Kinases
Rho/ROCK1: Ras Homolog Family/Protein Kinase N1
KLF4: Krüppel-Like Factor 4
CASP8: Caspase 8
CCDC134: Coiled-Coil Domain Containing 134
MAPK: Mitogen-Activated Protein Kinase
BMI: Body Mass Index
TNM: Tumor, Node, Metastasis
