# Supplementary material for: Tumor-derived EV miRNA signatures surpass total EV miRNA in supplementing mammography for precision breast cancer diagnosis
Source: Theranostics. 2024 Oct 7;14(17):6587–604. doi: 10.7150/thno.99245 (PMC11519808; doi:10.7150/thno.99245)
Supplement: Supplementary file 1 — Supplementary figures and tables. [file thnov14p6587s1.pdf]

Supplementary Information

Tumor-derived EV miRNA signatures surpass total EV miRNA in supplementing mammography for precision breast cancer diagnosis

Young Kim[†], Jee Ye Kim[†], Sol Moon, Hyojung Lee, Suji Lee, Joon Ye Kim, Min Woo Kim[*], Seung Il Kim[*]

Department of Surgery, Yonsei University College of Medicine, Seoul 03722, Republic of Korea

[†] These authors contributed equally to this work.

[*] Corresponding Authors:

Seung Il Kim and Min Woo Kim

Division of Breast Surgery, Department of Surgery, Yonsei University College of Medicine, 50-1 Yonsei-ro, Seodaemun-gu, Seoul 03722, Republic of Korea

Tel. +82-2-2228-0900; E-mails: SKIM@yuhs.ac and minwookim@yuhs.ac

This file includes:

Figures S1–S7

Tables S1–S5

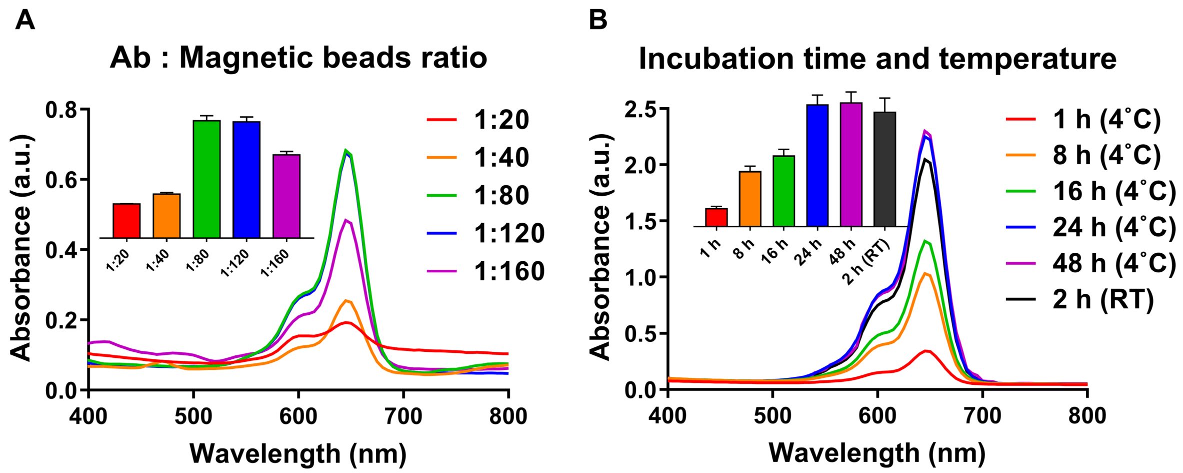

Figure S1. Optimization of antibody (Ab) to magnetic beads (Mg beads) ratio and incubation time for the isolation of breast cancer-derived extracellular vesicles (BEVs) using MDA-MB-231 EVs. (A) Absorbance spectra showing the effect of different Ab to Mg beads ratios on BEV isolation. The inset bar graph presents peak absorbance values at specified wavelengths for each ratio. (B) Absorbance spectra illustrating the impact of various incubation times on BEV isolation. The inset bar graph shows peak absorbance values at different time points.

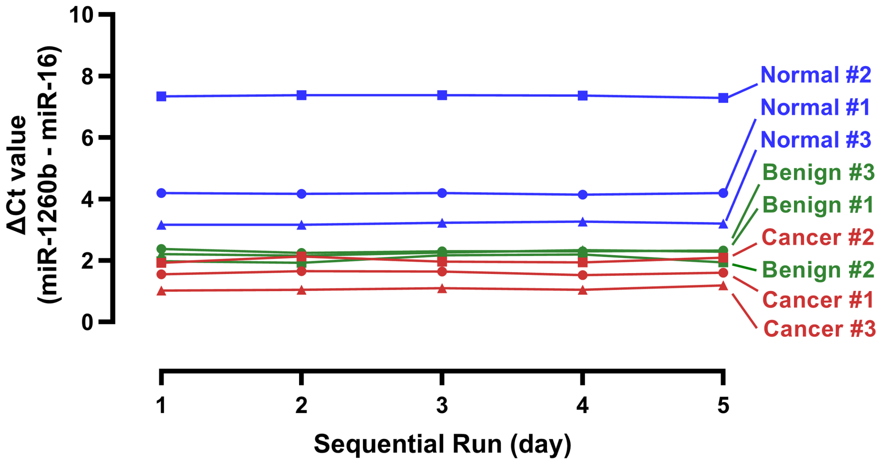

Figure S2. Time series plot showing the reproducibility of miRNA ΔCt values over 5 days across different sample types, run by a single experimenter. The graph shows the ΔCt values of miR-1260b (y-axis) measured sequentially over 5 days (x-axis) for various sample groups, including normal controls (blue lines), benign patients (green lines), and cancer patients (red lines), with three samples per group.

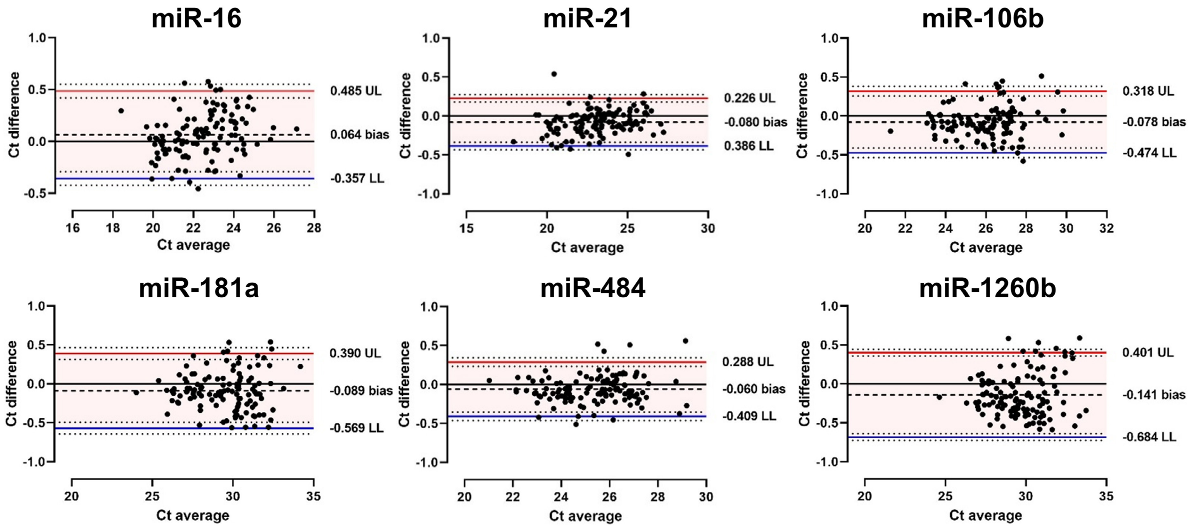

Figure S3. Bland-Altman plots showing the repeatability of Ct values for various miRNAs (miR-16, miR-21, miR-106b, miR-181a, miR-484, and miR-1260b) in cancer patients (n=120). Each plot displays the difference in Ct values (y-axis) versus the average Ct values (x-axis) for paired measurements. The dashed black line represents the mean difference (bias), while the solid red and blue lines indicate the upper limits (UL) and lower limits (LL) of agreement, respectively, based on a 95% confidence interval.

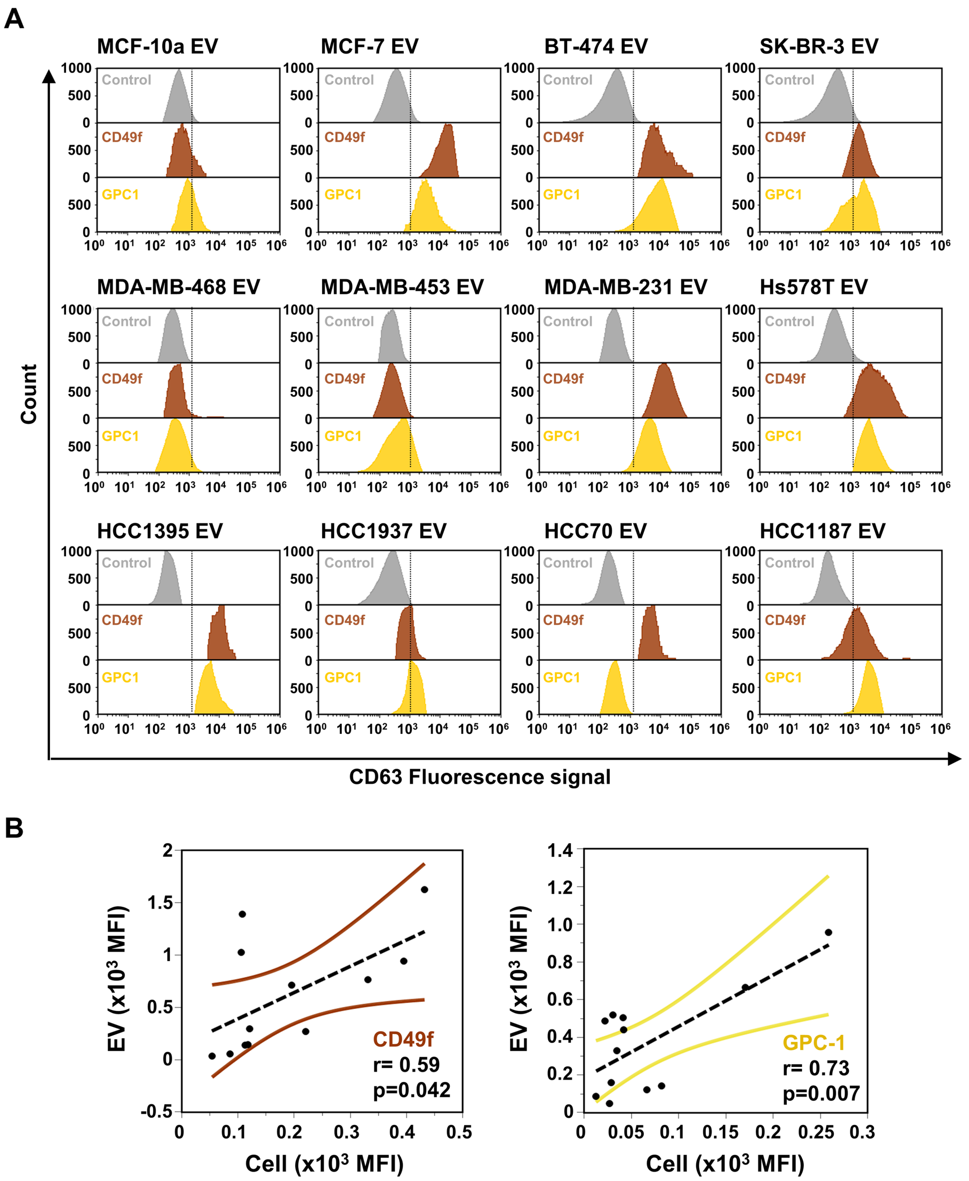

Figure S4. Evaluation of BEV surface protein marker expression. (A) Flow cytometry analysis of CD49f (brown) and GPC-1 (yellow) on EVs from 11 breast cancer cell lines and one control cell line (MCF10a). CD63-PE-Cy7 was used as a general EV marker for quantification. Histograms display the fluorescence intensity of BEVs for each marker, with control beads (gray) as a comparison. (B) Correlation between EV and cell surface marker expression for CD49f (brown) and GPC-1 (yellow). The Pearson correlation coefficient (r) and corresponding p-value indicate the significance of the correlation.

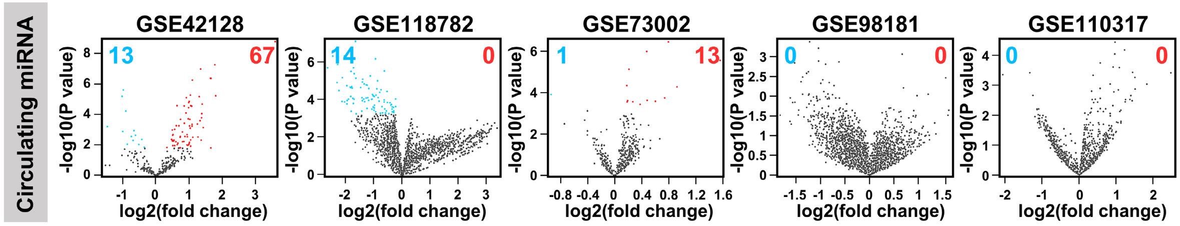

Figure S5. Volcano plots of differentially expressed microRNAs (miRNAs) in the blood of breast cancer patients using public datasets, including GSE42128, GSE118782, GSE73002, GSE98181, and GSE110317. Each plot compares the expression levels of miRNAs in breast cancer patients to normal controls, depicting log2 fold changes against the negative log10 of the p-value. Red dots indicate miRNAs with higher expression in patients, blue dots represent miRNAs with lower expression, and gray dots denote miRNAs with non-significant differential expression. The number of miRNAs showing high and low expression is annotated in each plot for enhanced clarity.

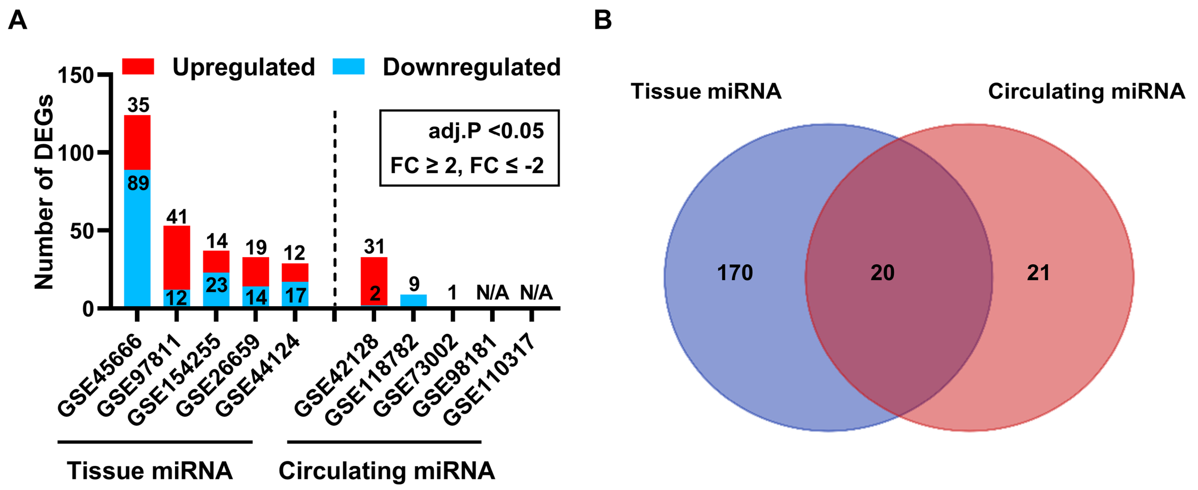

Figure S6. Analysis of differentially expressed microRNAs (miRNAs) in tissue and blood samples. (A) Bar chart illustrating the number of differentially expressed genes (DEGs) in various public datasets. Red bars represent upregulated miRNAs, and blue bars represent downregulated miRNAs in breast cancer tissue samples compared to controls. Datasets are indicated below each pair of bars, with the total number of upregulated and downregulated miRNAs labeled. The criteria for differential expression include an adjusted p-value < 0.05 and a fold change (FC) ≥ 2, FC ≤ -2. (B) Venn diagram displaying the overlap between differentially expressed tissue miRNAs and circulating miRNAs. The numbers indicate unique and shared miRNAs between the two sample types, highlighting the extent of expression correlation and potential biomarkers for breast cancer.

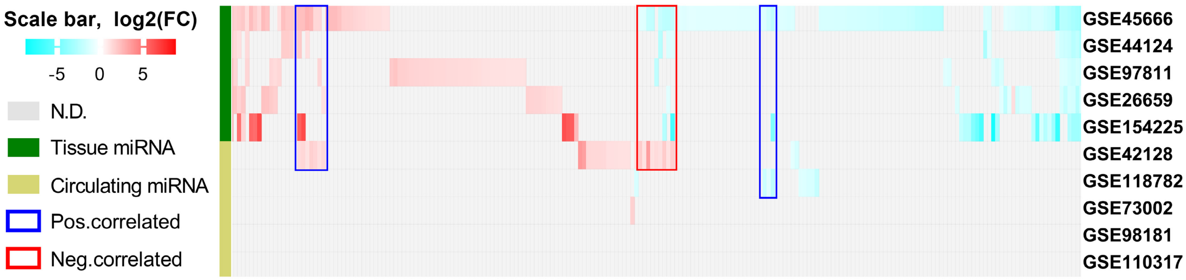

Figure S7. Comparative analysis of differentially expressed microRNAs (miRNAs) across multiple datasets. The heatmap visualizes the differential expression levels of miRNAs in tissue samples (green) and circulating miRNAs (olive) across multiple datasets. The color scale represents log2(fold change), with red indicating upregulation, cyan indicating downregulation, and gray indicating not detected (N.D.). Blue and red boxes within the heatmap highlight notable regions of positive and negative correlations between tissue and circulating miRNAs, respectively.

Table S1. The information of public datasets

GSE ID	Cohorts (BC *vs* N)	Total	Samples	Platform	Year	Ref
**Tissue miRNA analysis**						
**GSE26659**	77 *vs* 17	94	BC biopsies & normal tissues from mammoplastic reductions	Agilent human miRNA microarray (V2)	2012	[1]
**GSE45666**	101 *vs* 15	116	Breast tumor tissues	Agilent human miRNA microarray (V3)	2013	[2]
**GSE44124**	50 *vs* 30	80	Breast tumor tissues	Agilent human miRNA microarray (V3)	2014	[3]
**GSE97811**	45 *vs* 16	61	Breast tumor tissues	3D-Gene® Human miRNA (V21)	2017	[4]
**GSE154255**	10 *vs* 10	20	Breast tumor tissues	Agilent human miRNA microarray (V19)	2023	N/A
**Circulating miRNA analysis**						
**GSE42128**	32 *vs* 22	54	Serum samples from BC patients & healthy individuals	Exiqon miRCURY LNA microRNA array	2013	[5]
**GSE73002**	1,290 *vs* 54	1,344	Serum samples from BC patients & patients with benign breast disease	3D-Gene® Human miRNA (V20)	2016	[6]
**GSE98181**	24 *vs* 24	48	Serum samples from BC patients & cancer-free women	Affymetrix Multispecies miRNA	2018	[7]
**GSE118782**	30 *vs* 10	40	Plasma samples from BC patients & healthy women	Affymetrix Multispecies miRNA	2019	N/A
**GSE110317**	921 *vs* 37	958	Serum samples from BC patients & patients with benign breast disease	3D-Gene® Human miRNA (V21)	2023	[8]

* GSE, Gene Expression Omnibus Series; BC, breast cancer; N, normal control; N/A, not available

References for Table S1

1. Cimino D, De Pittà C, Orso F, Zampini M, Casara S, Penna E, et al. miR148b is a major coordinator of breast cancer progression in a relapse-associated microRNA signature by targeting ITGA5, ROCK1, PIK3CA, NRAS, and CSF1. Faseb J. 2013; 27: 1223-35.

2. Lee CH, Kuo WH, Lin CC, Oyang YJ, Huang HC, Juan HF. MicroRNA-Regulated Protein-Protein Interaction Networks and Their Functions in Breast Cancer. Int J Mol Sci. 2013; 14: 11560-606.

3. Feliciano A, Castellvi J, Artero-Castro A, Leal JA, Romagosa C, Hernández-Losa J, et al. miR-125b Acts as a Tumor Suppressor in Breast Tumorigenesis via Its Novel Direct Targets ENPEP, CK2-α, CCNJ, and MEGF9. Plos One. 2013; 8.

4. Hironaka-Mitsuhashi A, Matsuzaki J, Takahashi RU, Yoshida M, Nezu Y, Yamamoto Y, et al. A tissue microRNA signature that predicts the prognosis of breast cancer in young women. Plos One. 2017; 12.

5. Chan M, Liaw CS, Ji SM, Tan HH, Wong CY, Thike AA, et al. Identification of circulating microRNA signatures for breast cancer detection. Clin Cancer Res. 2013; 19: 4477-87.

6. Shimomura A, Shiino S, Kawauchi J, Takizawa S, Sakamoto H, Matsuzaki J, et al. Novel combination of serum microRNA for detecting breast cancer in the early stage. Cancer Sci. 2016; 107: 326-34.

7. Farina NH, Ramsey JE, Cuke ME, Ahern TP, Shirley DJ, Stein JL, et al. Development of a predictive miRNA signature for breast cancer risk among high-risk women. Oncotarget. 2017; 8: 112170-83.

8. Shiino S, Matsuzaki J, Shimomura A, Kawauchi J, Takizawa S, Sakamoto H, et al. Serum miRNA-based Prediction of Axillary Lymph Node Metastasis in Breast Cancer. Clin Cancer Res. 2019; 25: 1817-27.

Table S2. Logistic regression comparison for discovering miRNA combinations

	Combination			Logistic regression equation	Sensitivity	Specificity	AUC	SE	95% CI
1	miR-21	miR-106b		-4.181+0.748*LN(miR_21)+1.998*LN(miR_106b)	81.58%	78.02%	0.881	0.024	0.828 to 0.922
2	miR-21	miR-181a		-1.987+0.813*LN(miR_21)+0.351*LN(miR_181a)	70.18%	78.02%	0.806	0.03	0.745 to 0.857
3	miR-21	miR-484		-3.003+0.677*LN(miR_21)+1.230*LN(miR_484)	78.07%	75.82%	0.846	0.027	0.789 to 0.893
4	miR-21	miR-1260b		-1.650+0.904*LN(miR_21)0.013*LN(miR_1260b)	71.93%	80.22%	0.81	0.03	0.749 to 0.861
5	miR-106b	miR-181a		-3.390+2.158*LN(miR_106b)+0.298*LN(miR_181a)	74.56%	78.02%	0.837	0.028	0.779 to 0.884
6	miR-106b	miR-484		-4.25+1.847*LN(miR_106b)+1.180*LN(miR_484)	79.82%	78.02%	0.865	0.025	0.810 to 0.909
7	miR-106b	miR-1260b		-3.340+2.240*LN(miR_106b)+0.191*LN(miR_1260b)	77.19%	74.73%	0.838	0.028	0.781 to 0.886
8	miR-181a	miR-484		-2.636+0.379*LN(miR_181a)+1.489*LN(miR_484)	72.81%	68.13%	0.808	0.03	0.747 to 0.859
9	miR-181a	miR-1260b		-1.533+0.704*LN(miR_181a)+0.335*LN(miR_1260b)	68.42%	65.93%	0.732	0.035	0.666 to 0.792
10	miR-484	miR-1260b		-2.413+2.035*LN(miR_484)-0.225*LN(miR_1260b)	73.68%	72.53%	0.809	0.03	0.749 to 0.861
11	miR-21	miR-106b	miR-181a	-4.199+0.744*LN(miR_21)+1.973*LN(miR_106b)+0.032*LN(miR_181a)	79.82%	78.02%	0.88	0.027	0.828 to 0.921
12	miR-21	miR-106b	miR-484	-4.780+0.630*LN(miR_21)+1.716*LN(miR_106b)+0.822*LN(miR_484)	82.46%	80.22%	0.893	0.022	0.842 to 0.931
13	miR-21	miR-106b	miR-1260b	-4.14+0.809*LN(miR_21)+2.031*LN(miR_106b)-0.117*LN(miR_1260b)	81.58%	80.22%	0.881	0.024	0.829 to 0.922
14	miR-21	miR-181a	miR-484	-3.087+0.647*LN(miR_21)+0.140*LN(miR_181a)+1.180*LN(miR_484)	74.56%	73.63%	0.844	0.027	0.787 to 0.891
15	miR-21	miR-181a	miR-1260b	-1.983+0.817*LN(miR_21)+0.352*LN(miR_181a)-0.007*LN(miR_1260b)	70.18%	78.02%	0.806	0.03	0.745 to 0.858
16	miR-21	miR-484	miR-1260b	-3.296+0.886*LN(miR_21)+1.860*LN(miR_484)-0.567*LN(miR_1260b)	78.95%	79.12%	0.865	0.025	0.810 to 0.908
17	miR-106b	miR-181a	miR-484	-4.262+1.795*LN(miR_106b)+0.084*LN(miR_181a)+1.149*LN(miR_484)	79.82%	78.02%	0.864	0.025	0.810 to 0.908

18	miR-106b	miR-181a	miR-1260b			-3.516+2.057*LN(miR_106b)+0.267*LN(miR_181a)+0.171*LN(miR_1260b)	75.44%	75.82%	0.842	0.027	0.784 to 0.889
19	miR-106b	miR-484	miR-1260b			-4.286+1.855*LN(miR_106b)+1.450*LN(miR_484)-0.206*LN(miR_1260b)	80.70%	76.92%	0.87	0.025	0.816 to 0.913
20	miR-181a	miR-484	miR-1260b			-2.659+0.372*LN(miR_181a)+1.771*LN(miR_484)-0.211*LN(miR_1260b)	72.81%	71.43%	0.815	0.029	0.755 to 0.866
21	miR-21	miR-106b	miR-181a	miR-484		-4.763+0.640*LN(miR_21)+1.751*LN(miR_106b)-0.083*LN(miR_181a)+0.848*LN(miR_484)	82.46%	81.32%	0.892	0.022	0.841 to 0.931
22	miR-21	miR-106b	miR-181a	miR-1260b		-4.166+0.805*LN(miR_21)+2.014*LN(miR_106b)+0.040*LN(miR_181a)-0.118*LN(miR_1260b)	80.70%	80.22%	0.881	0.024	0.829 to 0.922
23	miR-21	miR-106b	miR-484	miR-1260b		-5.039+0.822*LN(miR_21)+1.677*LN(miR_106b)+1.407*LN(miR_484)-0.494*LN(miR_1260b)	84.21%	82.42%	0.906	0.021	0.857 to 0.942
24	miR-21	miR-181a	miR-484	miR-1260b		-3.336+0.867*LN(miR_21)+0.080*LN(miR_181a)+1.818*LN(miR_484)-0.560*LN(miR_1260b)	78.07%	79.12%	0.864	0.025	0.809 to 0.908
25	miR-106b	miR-181a	miR-484	miR-1260b		-4.291+1.810*LN(miR_106b)+0.070*LN(miR_181a)+1.418*LN(miR_484)-+0.202*LN(miR_1260b)	80.70%	76.92%	0.87	0.025	0.816 to 0.913
26	miR-21	miR-106b	miR-181a	miR-484	miR-1260b	-5.027+0.841*LN(miR_21)1.737*LN(miR_106b)-0.133*LN(miR_181a)+1.466*LN(miR_484)-0.506*LN(miR_1260b)	85.09%	84.62%	0.905	0.021	0.856 to 0.941

Table S3. 2x2 contingency table for accuracy of miRNA signature

		**Positive**	**Negative**	**Total**
**miRNA signature**	**Elevated**	103	14	117
	**Not elevated**	17	77	94
	**Total**	120	91	211

**Clinical Performance Parameters**	**N = 211**	**95% CI**
**Clinical Sensitivity**	85.83%	78.3 - 91.5
**Clinical Specificity**	84.62%	75.5 - 91.3
**Likelihood Ratio Negative (LRN)**	0.17	0.11 - 0.27
**Likelihood Ratio Positive (LRP)**	5.58	3.43 - 9.09
**Area Under Curve (AUC)**	0.908	0.861 - 0.943

Table S4. Determination results of miRNA signatures for each subject according to mammography results (BI-RADS)

Mammography (BI-RADS)	Breast cancer patients			Patients with benign breast disease		
		miRNA Signature expression			miRNA Signature expression	
	**N (%)**	**Low, N (%)**	**High, N (%)**	**N (%)**	**Low, N (%)**	**High, N (%)**
0	27 (22.50)	3 (11.11)	24 (88.89)	14 (30.43)	13 (92.86)	1 (7.14)
1	4 (3.33)	0 (0.00)	4 (100.00)	3 (6.52)	3 (100.00)	0 (0.00)
2	6 (5.00)	1 (16.67)	5 (83.33)	5 (10.87)	5 (100.00)	0 (0.00)
3	18 (15.00)	2 (11.11)	16 (88.89)	4 (8.70)	3 (75.00)	1 (25.00)
4	32 (26.67)	9 (28.13)	23 (71.88)	6 (13.04)	5 (83.33)	1 (16.67)
5	28 (23.33)	0 (0.00)	28 (100.00)	-		
N/A	5 (4.17)	2 (40.00)	3 (60.00)	14 (30.43)	12 (85.71)	2 (14.29)

Table S5. Clinical sensitivity of mammography, miRNA signature, and combined assay according to breast density

**Breast Density**	**N**	**miR Signature**	***p* value**	**Sensitivity (%)**		
				**MAMMO**	**miR Signature**	**Combined assay**
**Each grade**						
**A (Fatty)**	4	0.650 ± 0.254		75.00 (3/4)	75.00 (3/4)	100.00 (4/4)
**B (Scattered)**	14	0.864 ± 0.211	0.382	28.57 (4/14)	92.86 (13/14)	100.00 (14/14)
**C (Heterogeneously dense)**	84	0.790 ± 0.244		50.00 (42/84)	86.90 (73/84)	94.05 (79/84)
**D (Extremely dense)**	14	0.762 ± 0.243		78.57 (11/14)	85.71 (12/14)	100.00 (14/14)
**Non-dense breast *vs* Dense breast**						
**A, B**	18	0.816 ± 0.232		38.89 (7/18)	88.89 (16/18)	100.00 (18/18)
**C, D**	98	0.786 ± 0.242	0.58	54.08 (53/98)	86.73 (85/98)	94.90 (93/98)
**Total**	116	0.791 ± 0.240		51.72 (60/116)	87.07 (101/116)	95.69 (111/116)
